# Vibration of effects from diverse inclusion/exclusion criteria and analytical choices: 9216 different ways to perform an indirect comparison meta-analysis

**DOI:** 10.1186/s12916-019-1409-3

**Published:** 2019-09-16

**Authors:** Clément Palpacuer, Karima Hammas, Renan Duprez, Bruno Laviolle, John P. A. Ioannidis, Florian Naudet

**Affiliations:** 1 Centre d’Investigation Clinique INSERM 1414, Hôpital de Pontchaillou, 2 rue Henri le Guilloux, 35033 Rennes cedex 9, France; 2 Department of Biostatistics, Institut de Cancérologie de l’Ouest Centre René-Gauducheau, Saint-Herblain, France; 3 Department of Epidemiology and Biostatistics and Clinical Research, Assistance Publique-Hôpitaux de Paris, Hôpital Bichat Claude Bernard, Paris, France; 4 Inserm, CIC-EC 1425, Hôpital Bichat Claude Bernard, Paris, France; 5 Fondation Saint Jean de Dieu, Centre Hospitalier Dinan/St Brieuc, Dinan, France; 6 Department of Biological and Clinical Pharmacology and Pharmacovigilance, Rennes University Hospital, Rennes, France; 7 Laboratory of Experimental and Clinical Pharmacology, Rennes 1 University, Rennes, France; 8 Meta-Research Innovation Center at Stanford (METRICS), Stanford University, Stanford, CA USA; 9 Departments of Medicine, of Health Research and Policy, Biomedical Data Science, and Statistics, Stanford University, Stanford, CA USA

**Keywords:** Meta-analysis, Vibration of effect, Alcoholism, Nalmefene, Naltrexone

## Abstract

**Background:**

Different methodological choices such as inclusion/exclusion criteria and analytical models can yield different results and inferences when meta-analyses are performed. We explored the range of such differences, using several methodological choices for indirect comparison meta-analyses to compare nalmefene and naltrexone in the reduction of alcohol consumption as a case study.

**Methods:**

All double-blind randomized controlled trials (RCTs) comparing nalmefene to naltrexone or one of these compounds to a placebo in the treatment of alcohol dependence or alcohol use disorders were considered. Two reviewers searched for published and unpublished studies in MEDLINE (August 2017), the Cochrane Library, Embase, and ClinicalTrials.gov and contacted pharmaceutical companies, the European Medicines Agency, and the Food and Drug Administration. The indirect comparison meta-analyses were performed according to different inclusion/exclusion criteria (based on medical condition, abstinence of patients before inclusion, gender, somatic and psychiatric comorbidity, psychological support, treatment administered and dose, treatment duration, outcome reported, publication status, and risk of bias) and different analytical models (fixed and random effects). The primary outcome was the vibration of effects (VoE), i.e. the range of different results of the indirect comparison between nalmefene and naltrexone. The presence of a “Janus effect” was investigated, i.e. whether the 1st and 99th percentiles in the distribution of effect sizes were in opposite directions.

**Results:**

Nine nalmefene and 51 naltrexone RCTs were included. No study provided a direct comparison between the drugs. We performed 9216 meta-analyses for the indirect comparison with a median of 16 RCTs (interquartile range = 12–21) included in each meta-analysis. The standardized effect size was negative at the 1st percentile (− 0.29, favouring nalmefene) and positive at the 99th percentile (0.29, favouring naltrexone). A total of 7.1% (425/5961) of the meta-analyses with a negative effect size and 18.9% (616/3255) of those with a positive effect size were statistically significant (*p* < 0.05).

**Conclusions:**

The choice of inclusion/exclusion criteria and analytical models for meta-analysis can result in entirely opposite results. VoE evaluations could be performed when overlapping meta-analyses on the same topic yield contradictory result.

**Trial registration:**

This study was registered on October 19, 2016, in the Open Science Framework (OSF, protocol available at https://osf.io/7bq4y/).

**Electronic supplementary material:**

The online version of this article (10.1186/s12916-019-1409-3) contains supplementary material, which is available to authorized users.

## Background

Meta-analyses have become very popular and widely used by public health decision-makers, pharmaceutical companies, and clinicians in their day-to-day practice. Conventional meta-analyses only consider direct evidence from randomized controlled trials. However, there is an increasing interest in obtaining evidence from indirect comparisons to fill the gaps in comparative effectiveness research. Both direct and indirect comparisons can be considered in large-scale network meta-analyses that rank multiple treatments [1]. However, the production of systematic reviews and meta-analyses has reached epidemic proportions [2–4], and sometimes, overlapping meta-analyses on the same topic obtain divergent results [5]. Discordant meta-analyses on the same topic have been a common recurring theme for diverse clinical questions [6–13]. The discordance is often due to the differences in the way that the meta-analyses were conducted, e.g. the studies considered to be eligible; how searches are performed; the models used for data synthesis; and how results are interpreted. For example, it has been shown that the estimation of treatment outcomes in meta-analyses differs depending on the analytic strategy used [14]. However, each topic and each case may have its own special considerations that explain the discordance. It would be useful to develop a heuristic approach that can systematically and objectively assess the potential for obtaining discordant results in any meta-analysis topic.

Here, we propose applying the vibration of effect (VoE) concept as a tool for examining the spectrum of results that can be obtained in meta-analyses when different choices are made. VoE describes the extent to which an effect may change under multiple distinct analyses, such as different model specifications in epidemiological research [15–17].

As a case study, we explored VoE in a very concrete and controversial example with regulatory implications: the comparison of nalmefene and naltrexone in the treatment of alcohol use disorders. Nalmefene is a 6-methyl derivative of naltrexone [18]. Both are opioid antagonists [19]. Naltrexone is an approved treatment, historically used in the post-withdrawal maintenance of alcohol abstinence, whereas nalmefene has only recently been approved by the European Medicine Agency (EMA) [20] for the indication of reducing alcohol consumption, based on a posteriori analyses of pivotal trials performed on subgroups of patients with a high risk of drinking [21]. Because nalmefene was the first drug approved in this new indication, the phase III clinical programme did not compare this compound with naltrexone or another active comparator. However, several health authorities have highlighted the need for comparative efficacy data between the two drugs [22]. The only available comparison of the two drugs is provided by two indirect and conflicting meta-analyses. The first meta-analysis, funded by Lundbeck (manufacturer of nalmefene), found an advantage of nalmefene over naltrexone [23] with subgroup analyses on nalmefene RCTs compared with naltrexone RCTs as a whole. This methodological choice probably resulted in a violation of the similarity assumption, which is necessary for indirect meta-analyses [21, 24]. The second meta-analysis, performed by our team, did not find any significant difference between nalmefene and naltrexone [25]. Thus, a single analytical choice (subgroup analysis of the data versus full analysis set) affected the estimated effect size for treatment effect. We hypothesized that many other methodological choices concerning the study selection process and statistical analyses can easily modify the effect sizes obtained and the inferences made from indirect comparisons. We thus explored VoE in a large number of indirect meta-analyses to compare nalmefene and naltrexone in the reduction of alcohol consumption, using different methodological choices concerning inclusion/exclusion criteria and analytical models.

## Methods

### Design

A standard protocol was developed and registered on October 19, 2016, before the beginning of the study, in the Open Science Framework (OSF, protocol available at https://osf.io/7bq4y/).

### Eligibility criteria

All double-blind randomized controlled trials (RCTs) comparing nalmefene to naltrexone or one of these compounds to a placebo in the treatment of alcohol dependence (AD) or alcohol use disorders were included, regardless of other patient eligibility criteria, treatment modalities, or study duration. Study reports in English, French, German, Spanish, and Portuguese were considered.

### Search strategy and study selection process

Eligible studies were identified from PubMed/MEDLINE, the Cochrane Library, and Embase, including conference abstracts. Searches were initially conducted as part of a previous systematic review and meta-analysis that compared nalmefene, naltrexone, acamprosate, baclofen, and topiramate for the reduction of alcohol consumption [25]. The same algorithm was used for all electronic databases: “Nalmefene OR Baclofen OR Acamprosate OR Topiramate OR Naltrexone AND Alcohol” with the filter “Clinical Trial”. The last update of the search was performed in August 2017.

Two reviewers (CP and RD) independently reviewed the titles and abstracts of all citations identified by the literature search. Two reviewers (CP and KH) independently examined the full text of relevant studies. All disagreements were resolved by consensus or consultation with another reviewer (FN). Unpublished studies were also searched for by consulting the registries of ClinicalTrials.gov, the Food and Drug Administration, and the EMA. The authors were contacted for further information as necessary. If no response was obtained after the first request, they were re-contacted.

### Assessment of methodological quality

The risk of bias was assessed using the Cochrane Collaboration tool for assessing the risk of bias [26] for each RCT included in the study by two independent reviewers (CP and KH). All disagreements were resolved by consensus or consultation with another reviewer (FN).

### Data collection

A data extraction sheet, based on the *Cochrane Handbook for Systematic Reviews of Interventions* guidelines [27], was used to collect data from the RCTs. Data collection was performed by two reviewers (CP and KH). All disagreements were resolved by consultation with a third reviewer (FN). Suspected duplicate studies were compared to avoid integrating data from several reports on the same study. For each included study, information concerning the characteristics of the study [year, country, publication status (i.e. published or unpublished), outcomes reported], trial participants [age, gender, medical condition (i.e. alcohol dependence or alcohol use disorders), abstinence before the beginning of the study, somatic, or psychiatric comorbidity], and intervention [treatments, dose, route of administration and duration, and psychological support] was extracted.

### Assessment of vibration of effects

For each RCT, the treatment effect was calculated and expressed as the standardized mean difference (SMD, Hedges’ *g*) for the different consumption outcomes (i.e. quantity of alcohol consumed, frequency of drinking, and abstinence) [28]. For abstinence, the log (odds ratio) was calculated and converted into SMD (Hedges’ *g*) when the criterion reported in the study was the percentage of abstinent or relapsing subjects (i.e. binary outcomes) [28]. For direct comparisons, an estimate of the overall effect (summary measure) was calculated using both fixed and random effects models, with the inverse variance method. Indirect comparisons were performed using the graph theoretical method [29], a frequentist approach.

As a principal outcome, we explored the VoE of the indirect comparison between nalmefene and naltrexone. We computed the distribution of point estimates of effect sizes (ESs) and their corresponding *p* values under various analytical scenarios defined by the combination of methodological choices. These methodological choices (detailed in Table 1) were based on different inclusion/exclusion criteria (i.e. medical condition, abstinence of patients before inclusion, gender, somatic and psychiatric comorbidity, psychological support, treatment administered and dose, treatment duration, outcome reported, publication status, or risk of bias) and different analytical models (i.e. fixed or random effects). A negative ES favoured nalmefene. Meta-analyses were considered to be statistically significant if the ES was associated with a *p* value < 0.05. The presence of a “Janus effect” was investigated by calculating the 1st and 99th percentiles of the distribution of the ES [16]. A Janus effect is defined as an ES which is in the opposite direction between the 1st and 99th percentiles of meta-analyses. It demonstrates the presence of substantial VoE [16]. In addition, we computed the distribution of the *I*^2^ indices and the *p* values of the test for heterogeneity (i.e. Cochran’s *Q* test) calculated for each scenario. Heterogeneity was considered to be statistically significant if the *p* value of the *Q* test was < 0.10.

**Table 1:** Definition of the different methodological choices and number of possible analytical scenarios

Category	Criteria	Number of possibilities
Medical condition	Inclusion of all studies (AUDs and/or AD) Exclusion of studies including patients with AUDs	2
Abstinence^†^	Inclusion of all studies (abstinent or non-abstinent patients) Exclusion of studies requiring a minimum period of abstinence of 5 days or more before the beginning of the study	2
Gender	Inclusion of all studies (mixed gender, males only or females only) Exclusion of studies with males or females only	2
Somatic comorbidity	Inclusion of all studies (patients with or without systematic somatic comorbidities) Exclusion of studies on patients with systematic somatic comorbidities (e.g. studies on patients with HIV)	2
Psychiatric comorbidity	Inclusion of all studies (patients with or without systematic psychiatric comorbidities) Exclusion of studies on patients with systematic psychiatric comorbidities (e.g. studies on depressed patients)	2
Psychological support	Inclusion of all studies (with or without psychological intervention) Exclusion of studies with no psychological intervention	2
Treatment and dose	Only approved dose and route of administration Approved dose and route of administration OR closest dose to the approved dose Maximum dose tested	3
Treatment duration	Inclusion of all studies, regardless of treatment duration Exclusion of studies with a treatment duration of less than 12 weeks	2
Outcome^‡^	Quantity of alcohol consumed* Frequency of drinking** Abstinence***	3
Publication	Published and unpublished studies (e.g. study reports, ClinicalTrials.gov) Exclusion of unpublished studies	2
Risk of bias	Inclusion of all studies, regardless of the risk of selective outcome reporting Exclusion of studies with a high risk of selective outcome reporting	2
Analysis	Fixed effect model Random effect model	2
Total of possible combinations		9216

^†^The choice of the 5-day cut-off was based on our previous meta-analysis [25]

^‡^If there were several outcomes for quantity consumed, frequency of drinking, or abstinence reported in the same study, only one criterion of each type was collected

*Outcomes for the quantity of alcohol consumed were extracted in this order of preference: (1) total alcohol consumption, (2) number of drinks per day, (3) number of drinks per drinking day, and (4) alcohol consumption per drinking day

**Frequency of drinking outcomes was extracted in this order of preference: (1) number of heavy drinking days, (2) percentage of heavy drinking days, and (3) percentage of drinking days

***Abstinence outcomes were extracted in this order of preference: (1) number of abstinent days, (2) percentage of abstinent days, (3) percentage of abstinent subjects, and (4) percentage of relapsing subjects

The same approach was used to analyse the secondary outcomes: VoE of the direct comparison of nalmefene to placebo and VoE of the direct comparison of naltrexone to placebo. For these analyses, a negative ES favoured the experimental treatment (i.e. nalmefene or naltrexone).

All analyses were performed using R [30] and the metagen [31] and netmeta [32] libraries. The results are presented according to the Preferred Reporting Items for Systematic Reviews and Meta-Analyses (PRISMA) format [33] and its extension for network meta-analyses [34]. The data and code are shared on the Open Science Framework (available at https://osf.io/skv2h/).

### Changes to the initial protocol

As stated a priori in the protocol, we expected that several scenarios would not be feasible, depending on data availability, and some were therefore modified. Although we initially planned to collect only the consumption outcomes to assess VoE (i.e. quantity of alcohol consumed and frequency of drinking), we finally decided to extract abstinence outcomes as well, as many RCTs that assessed the efficacy of naltrexone for post-withdrawal maintenance of abstinence only reported extractable data on abstinence, and not consumption outcomes. Introduction of this new choice made it difficult to impute missing data at the patient level using imputation methods, and we suppressed the analytical scenario for which the meta-analyses were performed with imputed data. We also planned to conduct meta-analyses with all RCTs versus RCTs with treatment durations of 5 months or more. We changed the cut-off to 3 months as there were few RCTs with treatment durations of 5 months or more (especially for naltrexone trials). Furthermore, the impact of the risk of bias on the treatment effect was only assessed for the risk of selective outcome reporting and not the risk of incomplete outcome data, as almost all included RCTs were at high risk of incomplete outcome data. Finally, the VoE was not assessed according to patient age, language of publication, or subgroup analysis on patients with a high risk of drinking, because no RCT including minor patients was included in the study; all studies were published in English, except one (published in Portuguese but still included in the analyses even if only studies in English, French, and Spanish were to be retrieved initially); and no extractable data from the subgroup analyses on patients with a high risk of drinking were identified for the naltrexone studies. As part of the peer review process, we decided to perform a sensitivity analysis excluding meta-analyses with *I*^2^ > 25% and based on a fixed effect model, because these combinations could be considered inappropriate to do.

## Results

**Fig. 1:**
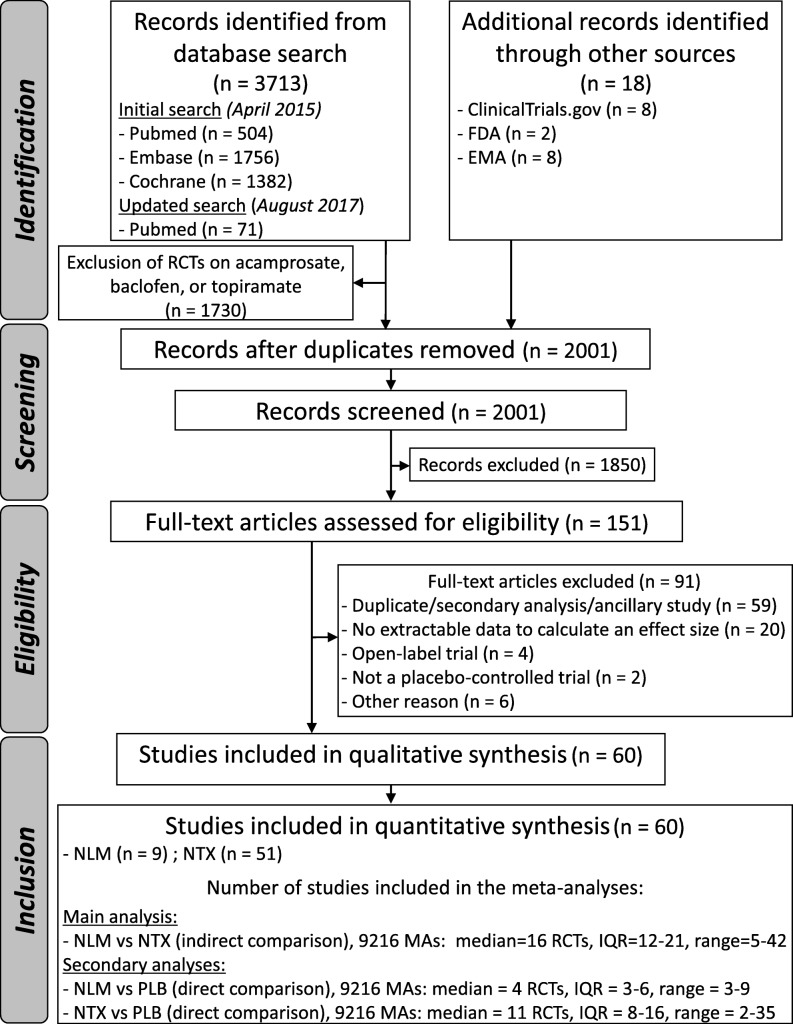
Flow diagram

After adjusting for duplicates, we identified a total of 2001 citations. After the first round of selection based on the titles and abstracts, the full texts of 151 articles were assessed for eligibility. We excluded 71 papers, and 20 articles provided no data with which to calculate an effect size on any relevant outcomes. References of the articles excluded after the review of the full text are listed in Additional file 1: References S1. A flowchart detailing the study selection process is shown in Fig. 1.

### Study characteristics and risk of bias within studies

Nine nalmefene versus placebo RCTs [35–41] and 51 naltrexone versus placebo RCTs [42–89] were considered eligible for the analyses. No study provided a direct comparison between nalmefene and naltrexone. The main characteristics of the included studies are summarized in Additional file 1: Table S1. The assessment of the risk of bias is reported in Additional file 1: Figure S1. One article was published in Portuguese [50]. Several studies were unpublished: data of 2 unpublished nalmefene RCTs (CPH-101-0399, CPH-101-0701) were provided by access to the document service of the EMA, and data of 3 naltrexone RCTs were recovered from ClinicalTrials.gov (NCT00667875, NCT01625091, and NCT00302133). No study was conducted on minor patients.

### Vibration of effects

The distribution of the studies according to each methodological choice is presented in Table 2. We performed 9216 overlapping meta-analyses for the indirect comparison of nalmefene to naltrexone (resulting in 3856 different RCT combinations), the direct comparison of nalmefene to placebo (resulting in 86 different RCT combinations), and the direct comparison of naltrexone to placebo (resulting in 1988 different RCT combinations).

**Table 2:** Distribution of the studies according to each possible methodological choice

Category	Nalmefene (*n* = 9)	Naltrexone (*n* = 51)
Medical condition		
AD only	6 (66.7%)	33 (64.7%)
AUDs	3 (33.3%)	18 (35.3%)
Abstinence		
< 5 days	9 (100%)	34 (66.7%)
≥ 5 days	0 (0.0%)	17 (33.3%)
Gender		
Mixed	9 (100%)	39 (76.5%)
Males or females only	0 (0.0%)	12 (23.5%)
Systematic somatic comorbidity		
No	9 (100%)	48 (94.1%)
Yes	0 (0.0%)	3 (5.9%)
Systematic psychiatric comorbidity		
No	9 (100%)	39 (76.4%)
Yes	0 (0.0%)	12 (23.5%)
Psychological support		
No	1 (11.1%)	4 (7.8%)
Yes	8 (88.9%)	47 (92.2%)
Treatment and dose		
Approved dose and route of administration		
No	6 (66.7%)	14 (27.5%)
Yes	3 (33.3%)	37 (72.5%)
Maximum dose tested		
No	8 (88.9%)	50 (98.0%)
Yes	1 (11.1%)	1 (2.0%)
Treatment duration		
≥ 12 weeks	9 (100%)	41 (80.4%)
< 12 weeks	0 (0.0%)	10 (19.6%)
Outcome reported		
Quantity of alcohol consumed		
No	0 (0.0%)	21 (41.2%)
Yes	9 (100%)	30 (58.8%)
Frequency of drinking		
No	2 (22.2%)	16 (31.4%)
Yes	7 (77.8%)	35 (68.6%)
Abstinence		
No	0 (0.0%)	18 (35.3%)
Yes	9 (100%)	33 (64.7%)
Publication		
Published	7 (77.8%)	48 (94.1%)
Unpublished	2 (22.2%)	3 (5.9%)
Risk of bias		
High risk of selective outcome reporting	2 (22.2%)	5 (9.8%)
Unclear or low risk of selective outcome reporting	7 (77.8%)	46 (90.2%)

Numbers are presented with their corresponding percentage

*AD* alcohol dependence, *AUD* alcohol use disorder

### Main analysis

**Fig. 2:**
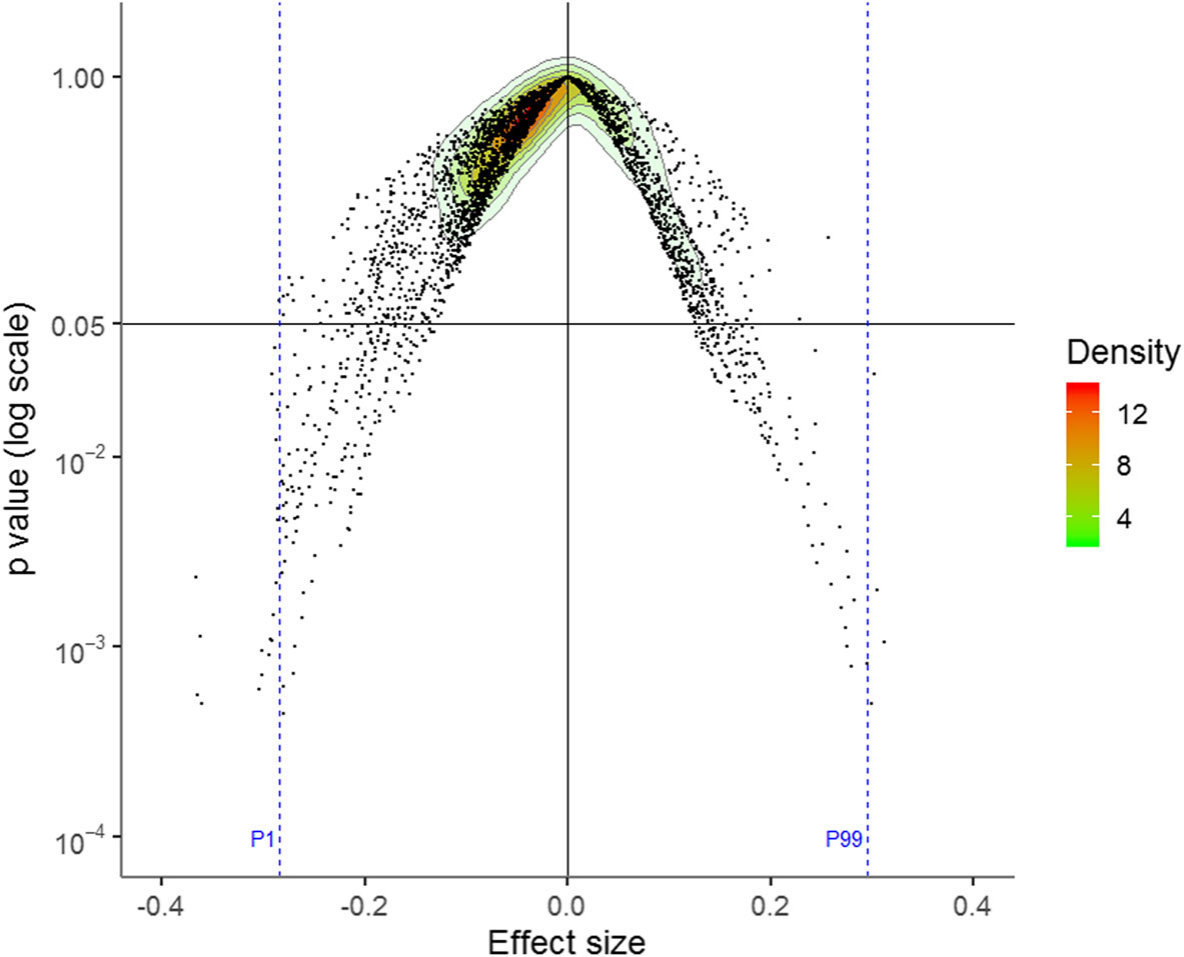
Vibration of effects for the indirect comparison of nalmefene to naltrexone. A negative effect size favours nalmefene, whereas a positive effect size favours naltrexone. The points represent the meta-analyses. The colours represent the densities

A median of 16 RCTs [interquartile range (IQR) = 12–21] were included in the meta-analyses for the indirect comparison (Fig. 1). The distribution of the ES ranged from − 0.37 to 0.31, with a median of − 0.04. The ES was negative for the 1st percentile (− 0.29) and positive for the 99th percentile (0.29) (Fig. 2), indicating the presence of a Janus effect, with some meta-analyses showing a statistically significant superiority of nalmefene over naltrexone, whereas others showed the opposite effect. A total of 7.1% (425/5961) of the meta-analyses with a negative ES (i.e. in favour of nalmefene) and 18.9% (616/3255) of the meta-analyses with a positive ES (i.e. in favour of naltrexone) were statistically significant (*p* < 0.05). An example of 2 meta-analyses with contradictory results is presented in Additional file 1: Table S2-S3. Concerning heterogeneity, the median of the *I*^2^ index was 14% (IQR = 0–42%), and the *p* value of Cochran’s *Q* test was < 0.10 for 31.4% (2896/9216) of the meta-analyses (Additional file 1: Figure S2). A similar VoE was found in the sensitivity analysis excluding the meta-analyses with *I*^2^ > 25% and based on a fixed effect model, with a negative ES for the 1st percentile (− 0.28) and a positive ES for the 99th percentile (0.30) (Additional file 1: Figure S3).

### Secondary analyses

**Fig. 3:**
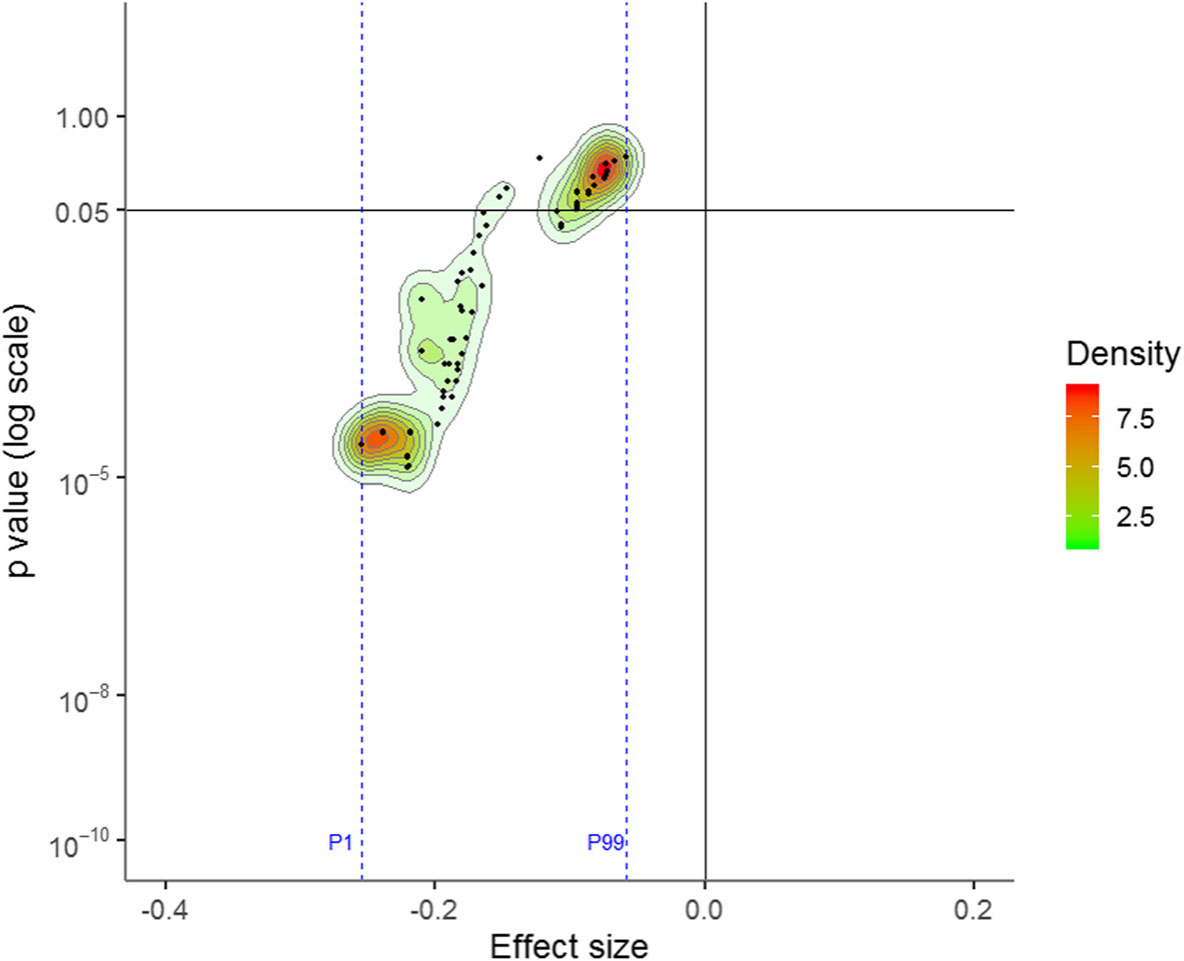
Vibration of effects for the direct comparison of nalmefene to placebo. A negative effect size favours nalmefene, whereas a positive effect size favours the placebo. The points represent the meta-analyses. The colours represent the densities

None of the meta-analyses performed on nalmefene RCTs favoured the placebo. The ES ranged from − 0.25 to − 0.06, with a median of − 0.19. The ES was negative for both the 1st percentile and 99th percentiles (− 0.25 and − 0.06), and there was no Janus effect (Fig. 3). A total of 67.4% (6208/9216) of the meta-analyses were statistically significant. The heterogeneity was generally small (median of *I*^2^ = 0%, IQR = 0–25%) (Additional file 1: Figure S4), and the *p* value of Cochran’s *Q* test was < 0.10 for 7.6% (704/9216) of the meta-analyses.

**Fig. 4:**
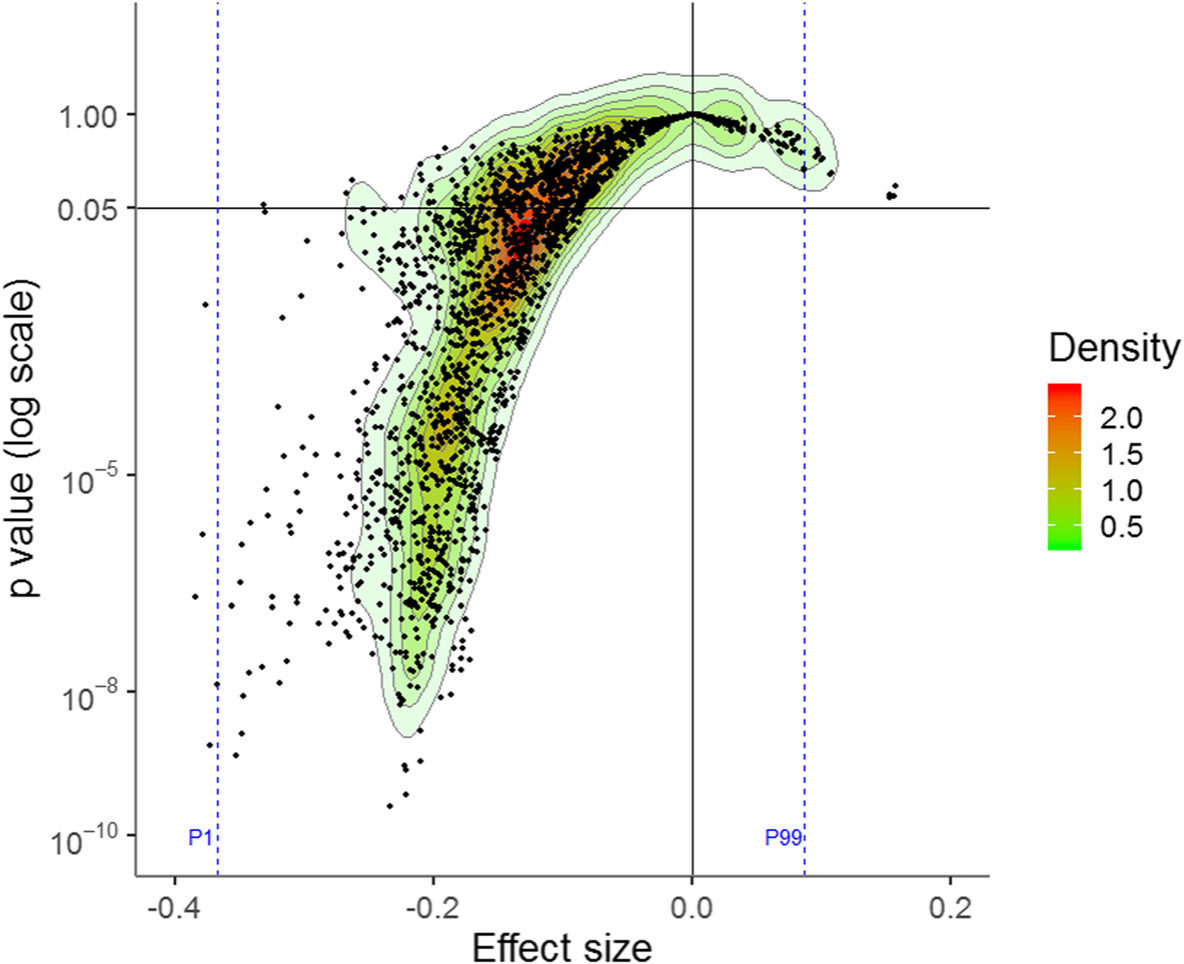
Vibration of effects for the direct comparison of naltrexone to placebo. A negative effect size favours naltrexone, whereas a positive effect size favours the placebo. The points represent the meta-analyses. The colours represent the densities

The meta-analyses performed on naltrexone versus placebo RCTs provided ESs ranging from − 0.38 to 0.16, with a median of − 0.16. Although the ESs favouring placebo over naltrexone never reached statistical significance, there was a Janus effect: the ES was in opposite directions between the 1st (ES = − 0.37) and 99th percentiles (ES = 0.09) of the meta-analyses (Fig. 4). Only 6.5% (602/9216) of the meta-analyses were associated with a positive ES. Heterogeneity (median of *I*^2^ = 23%, IQR = 3–48%) (Additional file 1: Figure S5) was higher than for direct comparisons of nalmefene to placebo. The *p* value of Cochran’s *Q* test was < 0.10 for 34.9% (3218/9216) of the meta-analyses.

## Discussion

### Statement of principal findings

VoE is a standardized method that can be used in any meta-analysis to systematically evaluate the breadth and divergence of the results, depending on the choices made in the selection of studies, based on various criteria, and the analytical model used. As a case study, we show extensive VoE in an indirect comparison of nalmefene to naltrexone, leading to contradictory results. Although most combinations yielded no evidence of a difference, some meta-analyses showed superiority of nalmefene, whereas others showed superiority of naltrexone. These two compounds have many similarities, and it is unlikely to expect a genuine difference [19]. When we considered direct comparisons against placebo, we observed less VoE for nalmefene than for naltrexone. Nalmefene is the most recent treatment option and has been the subject of two distinct but somewhat homogeneous development programmes [90], resulting in several studies with a similar design. In contrast, naltrexone is an older option with a myriad of pre- and post-approval RCTs conducted in very different settings.

### Strengths and weaknesses of this study

We recommend that a list of all possible major options should be made first when examining VoE in a meta-analysis. Nevertheless, we acknowledge that even the construction of such a list may itself be subject to unavoidable subjectivity. All the methodological choices we made to assess VoE in our case study corresponded to the criteria we considered to be easily “gameable”. Several may be clinically relevant, such as the exclusion of studies on abstinent patients or those on patients with a somatic or psychiatric comorbidity. Others are related to the literature search, such as the retrieval of unpublished studies. Most meta-analyses have difficulty unearthing unpublished studies, and publication bias [91] may affect the treatment ranking in indirect meta-analyses [92]. The relevance of other combinations may be debatable. For example, the use of fixed effect models in case of between-study heterogeneity is not considered statistically valid and would not be considered for publication. However, our sensitivity analysis that excluded meta-analyses that were considered inappropriate to do still found some VoE. It is difficult and subjective to judge the appropriateness of different combinations; the two illustrative examples (Additional file 1: Table S2-S3) demonstrate that contradictory meta-analyses are not necessarily inappropriate, per se. It is also likely that some datasets we combined violated the similarity assumption required for indirect comparisons. Dissimilar study results due to treatment effect modifiers may have led to some of the VoE we observed. In theory, positive and negative results from multiple meta-analyses are not necessarily contradictory if the inclusion criteria are so different that the results would apply to different research questions. However, in practice, the identification of treatment effect modifiers is very challenging [93], and it is sometimes very difficult and subjective to make a clear judgement on how much different methodological choices really define different research questions. Here, we tried to pre-emptively retain the choices that would not have altered the research question, and we minimized the possibility of making mutually exclusive methodological choices. Moreover, our study was based on a relatively limited number of methodological choices, which may underestimate the whole set of alternative scenarios, and the VoE could have been even greater. For example, we could have studied the VoE depending on whether the indirect comparisons were made using a Bayesian approach or a frequentist approach. Another potential source of VoE was not investigated, namely the choice of the source from which data from a study are extracted (e.g. published articles, study reports, ClinicalTrials.gov). It is possible that in other contexts, such as meta-analyses exploring drug safety, non-randomized studies may be included and add even more VoE, especially due to possible bias (e.g. indication bias) in the primary studies.

Indirect comparison meta-analyses may yield less VoE in other, less controversial fields, in which the results of studies are more homogeneous. Conversely, VoE may be more prominent in complex, heterogeneous meta-analyses, such as that of large networks with prominent inconsistency [94]. For example, it has been shown in network meta-analyses that even the consideration of which nodes are eligible and/or whether a placebo should be considered can already yield very different results [95]. In addition, some of the contradictory meta-analyses that are generated in the VoE exercise may not pass peer review, receive harsh criticism for their choices, or even be retracted after publication, as for a meta-analysis of acupuncture [96]. Therefore, we recommend that the choice of factors to consider in the VoE analyses should be realistic.

### Perspectives

VoE has already been described in the field of observational epidemiology [16, 97], but has been less explored in meta-analyses. Nevertheless, a previous study showed that it is possible to manipulate the effect sizes based on the discrepancies among multiple data sources (papers, clinical study reports, individual patient data) [98]. In this study, the overall result of the meta-analyses performed to assess the ES of gabapentin for the treatment of pain intensity switched from effective to ineffective and the overall result for the treatment of depression with quetiapine from medium to small, depending on the data source. In our study, cherry-picking results from each included RCT may have introduced VoE without changing either the trial inclusion criteria or the methods of meta-analysis.

There is a large body of literature on discordant meta-analyses. Indeed, the first widely known meta-analyses in medicine were probably those performed by opposing teams in the 1970s that found opposite results on the risk of gastrointestinal bleeding from steroids. Over the years, debate has often arisen within specific topics in which two or more meta-analyses on seemingly the same question reached different conclusions [6–13]. Discussion of the main reasons put forth for the discrepancy for each case, with careful clinical reasoning, is likely to continue being useful. VoE analysis offers a complementary systematic approach to evaluate the potential for a discrepancy in any meta-analysis, including large-scale meta-analyses. VoE offers a more generalized view of sensitivity analyses. Typically, some level of sensitivity analysis is commonly performed. For example, many/most meta-analyses may present side-by-side fixed and random effects models, as performed in our case study. Additional methodological choices may generate more sensitivity analyses related to the choice of outcome measure, handling of missing data, correction for potential bias, interdependence, etc. [99–102]. Sensitivity analyses based on clinical characteristics are also common, but usually, only a few such analyses are reported, if at all.

Although the evaluation of VoE is generally systematic and involves more extensive analysis than the sporadic sensitivity analyses typically performed in past meta-analyses, it still requires a priori determination of the factors considered to be most relevant for making choices in the conduct of a meta-analysis. In this respect, it is not as clinically agnostic as the all-subset method, in which all possible meta-analyses of all possible subsets of studies are explored for a given set of studies to be meta-analysed [103]. This method runs into computational difficulties with large meta-analyses. For example, application of the all-subset method for VoE in the current case study, with 51 and 9 trials, would result in 2^51^ and 2^9^ possible subsets, respectively, with 2^51 + 9^ = 2^60^ different indirect meta-analyses, i.e. 1,152,921,504,606,846,976 different indirect comparison meta-analyses to be performed, a number that is computationally absurd to explore and not clinically relevant.

Systematic reviews and meta-analyses (including indirect comparisons and more complex networks) are often considered to offer the highest level of evidence [104]. These studies have become so influential that they can shape guidelines and change clinical practice. However, their use has reached epidemic proportions, and published meta-analyses [104] and network meta-analyses [5] are subject to extensive overlap and potential redundancy. It has been argued that these studies could be used as key marketing tools when there are strong conflicts concerning which results are preferable to highlight. For example, numerous meta-analyses of antidepressants authored by or linked to the industry have been described previously [105]. Industry-linked studies almost never report any caveats about antidepressants in their abstracts. Conversely, it is a common practice in the industry to commission network meta-analyses to professional contracting companies, and thus, most are not registered a priori or published. A veto from industry is the most commonly stated reason for not having a publication plan for network meta-analyses [106]. VoE is a method that could be used to highlight selective reporting and controversial results.

The extent of redundant meta-analyses and wasted efforts may be reduced with protocol pre-registration, for example, with the PROSPERO database [107]. Nevertheless, registration does not provide the same guarantees for meta-analyses as for RCTs. For RCTs, registration is prospectively performed, before enrolment of the first patient. Conversely, meta-analyses are almost always retrospective (i.e. planned after the individual studies are completed), and even registration cannot prevent “cherry picking” of some methodological choices based on preliminary analyses of the existing data. The development of prospective meta-analyses could avoid these pitfalls, as in such meta-analyses, studies are identified, evaluated, and determined to be eligible before the results of any of the studies become known [27].

Even if meta-analysis protocols are thoroughly and thoughtfully designed, a number of analytical and eligibility choices still need to be made and many may be subjective. An applicable safeguard could be the a priori reviewing of protocols by independent experts and comities that might prevent meta-analyses of being gameable. In addition, VoE allows a systematic exploration of the influence of analytical and eligibility choices on the treatment effect. It appears to be a tool that is worth developing in different contexts, such as head-to-head, network, and individual patient data meta-analyses. Systematically exploring VoE in a large set of meta-analyses may provide a better sense of its relevance.

## Conclusions

Multiplication of overlapping meta-analyses may more frequently yield contradictory results that are difficult to interpret. Controversial and conflicting meta-analyses can result in the loss of credibility in the eyes of patients, the medical community, and the policy-makers. Efforts must be made to improve the reproducibility and transparency [108] of research and minimize VoE, whenever possible. In most circumstances, the most feasible approach would be to determine the magnitude of the potential risk of obtaining discrepant results, for which VoE could be a useful tool.

## Additional file

Additional file 1: **References S1.** References of articles excluded after review of full-texts. **Table S1.** Main characteristics of the included studies. **Figure S1.** Quality evaluation of studies included according to the Cochrane Collaboration tool for assessing risk of bias. **Table S2.** Analytical scenario resulting in superiority of nalmefene over naltrexone. **Table S3.** Analytical scenario resulting in superiority of naltrexone over nalmefene. **Figure S2.** Heterogeneity of the indirect comparison: nalmefene versus naltrexone. **Figure S3.** Sensitivity analysis excluding meta-analyses with *I*^2^ > 25% and based on a fixed effect model for the indirect comparison between nalmefene and naltrexone. **Figure S4.** Heterogeneity of the direct comparison: nalmefene versus placebo. Figure S5. Heterogeneity of the direct comparison: naltrexone versus placebo. **Checklist S1.** PRISMA checklist. (PDF 562 kb)

## Data Availability

The data and code are shared on the Open Science Framework (available at https://osf.io/skv2h/).

## Abbreviations

AD: Alcohol dependence
EMA: European Medicines Agency
ES: Effect size
RCT: Randomized control trial
SMD: Standardized mean difference
VoE: Vibration of effects
