# Supplementary material for: Vibration of effects from diverse inclusion/exclusion criteria and analytical choices: 9216 different ways to perform an indirect comparison meta-analysis
Source: BMC Med. 2019 Sep 16;17:174. doi: 10.1186/s12916-019-1409-3 (PMC6747755; doi:10.1186/s12916-019-1409-3)
Supplement: Supplementary file 1 — References S1. References of articles excluded after review of full-texts. Table S1. Main characteristics of the included studies. Figure S1. Quality evaluation of studies included according to the Cochrane Collaboration tool for assessing risk of bias. Table S2. Analytical scenario resulting in superiority of nalmefene over naltrexone. Table S3. Analytical scenario resulting in superiority of naltrexone over nalmefene. Figure S2. Heterogeneity of the indirect comparison: nalmefene versus naltrexone. Figure S3. Sensitivity analysis excluding meta-analyses with I2 > 25% and based on a fixed effect model for the indirect comparison between nalmefene and naltrexone. Figure S4. Heterogeneity of the direct comparison: nalmefene versus placebo. Figure S5. Heterogeneity of the direct comparison: naltrexone versus placebo. Checklist S1. PRISMA checklist. (PDF 562 kb) [file 12916_2019_1409_MOESM1_ESM.pdf]

Vibration of effects from diverse inclusion/exclusion criteria and analytical choices: 9,216 different ways to perform an indirect comparison meta-analysis

Clément Palpacuer, Karima Hammas, Renan Duprez, Bruno Laviolle, John P.A. Ioannidis, and Florian Naudet

Additional file 1

Contents of additional file 1

Additional file 1: References S1	References of articles excluded after review of full-texts	Page 3
Additional file 1: Table S1	Main characteristics of the included studies	Page 11
Additional file 1: Figure S1	Quality evaluation of studies included according to the Cochrane Collaboration tool for assessing risk of bias	Page 17
Additional file 1: Table S2	Analytical scenario resulting in superiority of nalmefene over naltrexone	Page 20
Additional file 1: Table S3	Analytical scenario resulting in superiority of naltrexone over nalmefene	Page 21
Additional file 1: Figure S2	Heterogeneity of the indirect comparison: nalmefene versus naltrexone	Page 22
Additional file 1: Figure S3	Sensitivity analysis excluding meta-analyses with I^2 > 25% and based on a fixed effect model for the indirect comparison between nalmefene and naltrexone	Page 23
Additional file 1: Figure S4	Heterogeneity of the direct comparison: nalmefene versus placebo	Page 24
Additional file 1: Figure S5	Heterogeneity of the direct comparison: naltrexone versus placebo	Page 25
Additional file 1: Checklist S1	PRISMA Checklist	Page 26

Additional file 1: References S1. References of articles excluded after review of full-texts

List of non-eligible articles

1 Ahmadi J, Ahmadi N. A double blind, placebo-controlled study of naltrexone in the treatment of alcohol dependence. Ger J Psychiatry 2002;5:85–9.

2 Ahmadi J, Kampman KM, Oslin DM, et al. Predictors of treatment outcome in outpatient cocaine and alcohol dependence treatment. Am J Addict 2009;18:81–6. doi:10.1080/10550490802545174

3 Allen JP, Sillamaukee P, Anton R. Contribution of carbohydrate deficient transferrin to gamma glutamyl transpeptidase in evaluating progress of patients in treatment for alcoholism. Alcohol Clin Exp Res 1999;23:115–20.

4 Anton RF, Moak DH, Waid LR, et al. Naltrexone plus cognitive-behavior therapy for alcoholism. 152nd Annu Meet Am Psychiatr Assoc Wash DC USA 15-20th May 1999 Published Online First: 1999.http://onlinelibrary.wiley.com/o/cochrane/clcentral/articles/469/CN-00278469/frame.html

5 Arias AJ, Gelernter J, Gueorguieva R, et al. Pharmacogenetics of naltrexone and disulfiram in alcohol dependent, dually diagnosed veterans. Am J Addict 2014;23:288–93. doi:10.1111/j.1521-0391.2014.12102.x

6 Armeli S, Feinn R, Tennen H, et al. The effects of naltrexone on alcohol consumption and affect reactivity to daily interpersonal events among heavy drinkers. Exp Clin Psychopharmacol 2006;14:199–208. doi:10.1037/1064-1297.14.2.199

7 Balldin J, Berglund M, Borg S, et al. The swedish naltrexone study, present results. 9th Congr Assoc Eur Psychiatr Cph Den 20-24th Sept 1998 Published Online First: 1998.http://onlinelibrary.wiley.com/o/cochrane/clcentral/articles/576/CN-00278576/frame.html

8 Baltieri DA, Daro FR, Ribeiro PL, et al. Effects of topiramate or naltrexone on tobacco use among male alcohol-dependent outpatients. Drug Alcohol Depend 2009;105:33–41. doi:10.1016/j.drugalcdep.2009.05.025

9 Baltieri DA, Daró FR, Ribeiro PL, et al. The role of alcoholic beverage preference in the severity of alcohol dependence and adherence to the treatment. Alcohol Fayettev N 2009;43:185–95. doi:10.1016/j.alcohol.2009.01.001

10 Baros AM, Latham PK, Anton RF. Naltrexone and cognitive behavioral therapy for the treatment of alcohol dependence: Do sex differences exist? Alcohol Clin Exp Res 2008;32:771–6. doi:10.1111/j.1530-0277.2008.00633.x

11 Batki SL, Leontieva L, Dimmock JA, et al. Negative symptoms are associated with less alcohol use, craving, and ‘high’ in alcohol dependent patients with schizophrenia. Schizophr Res 2008;105:201–7. doi:10.1016/j.schres.2008.06.020

12 Bohn MJ, Kranzler HR, Beazoglou D, et al. Naltrexone and brief counseling to reduce heavy drinking. Results of a small clinical trial. Am J Addict 1994;3:91–9.

13 Bold KW, Fucito LM, DeMartini KS, et al. Predictors of within-treatment drinking in a trial of targeted naltrexone versus placebo in young adults. Alcohol Clin Exp Res 2015;:76a. doi:10.1111/acer.12741

14 Bujarski S, O'Malley SS, Lunny K, et al. The effects of drinking goal on treatment outcome for alcoholism. J Consult Clin Psychol 2012;81:13–22. doi:10.1037/a0030886

15 Busch AC, Hetzel S, Brown RT. Pharmacotherapeutic intervention to improve treatment engagement among alcohol dependent veterans after hospital discharge. Alcohol Clin Exp Res 2015;:76a. doi:10.1111/acer.12741

16 Carroll K, Ziedonis D, O'Malley S, et al. Pharmacologic interventions for alcohol-and cocaine-abusing individuals. A pilot study of disulfiram vs. naltrexone. Am J Addict 1993;2:77–9.

17 Chick J. Opiate antagonists in the treatment of alcohol dependence: Results of a UK study CONFERENCE ABSTRACT. 9th Eur Coll Neuropsychopharmacol Congr Amst Neth 21st-25th Sept 1996 Published Online First: 1996.http://onlinelibrary.wiley.com/o/cochrane/clcentral/articles/418/CN-00279418/frame.html

18 Ciraulo DA, Dong Q, Silverman BL, et al. Early treatment response in alcohol dependence with extended-release naltrexone. J Clin Psychiatry 2008;69:190–5.

19 Connery H, Greenfield S, Livchits V, et al. Training and fidelity monitoring of alcohol treatment interventions integrated into routine tuberculosis care in Tomsk, Russia: the IMPACT Effectiveness Trial. Subst Use Misuse 2013;48:784–92. doi:10.3109/10826084.2013.793715

20 Cramer J, Rosenheck R, Kirk G, et al. Medication compliance feedback and monitoring in a clinical trial: predictors and outcomes. Value Health 2003;6:566–73.

21 Davidson D, Wirtz PW, Gulliver SB, et al. Naltrexone's suppressant effects on drinking are limited to the first 3 months of treatment. Psychopharmacol Berl 2007;194:1–10. doi:10.1007/s00213-007-0807-y

22 De Sousa A. A comparative study using Disulfiram and Naltrexone in alcohol-dependent adolescents. J Subst Use 2014;19:341–5. doi:10.3109/14659891.2013.813084

23 DeMartini KS, Gueorguieva R, Leeman RF, et al. Longitudinal findings from a randomized clinical trial of naltrexone for young adult heavy drinkers. J Consult Clin Psychol 2016;84:185–90. doi:10.1037/ccp0000053

24 Dolan SL, Rohsenow DJ, Martin RA, et al. Urge-specific and lifestyle coping strategies of alcoholics: relationships of specific strategies to treatment outcome. Drug Alcohol Depend 2012;128:8–14. doi:10.1016/j.drugalcdep.2012.07.010

25 Donovan DM, Anton RF, Miller WR, et al. Combined pharmacotherapies and behavioral interventions for alcohol dependence (The COMBINE Study): examination of posttreatment drinking outcomes. J Stud Alcohol Drugs 2007;69:5–13.

26 Farren CK, Scimeca M, Wu R, et al. A double-blind, placebo-controlled study of sertraline with naltrexone for alcohol dependence. Drug Alcohol Depend 2008;99:317–21. doi:10.1016/j.drugalcdep.2008.06.006

27 Feinn R, Tennen H, Cramer J, et al. Measurement and prediction of medication compliance in problem drinkers. Alcohol Clin Exp Res 2003;27:1286–92. doi:10.1097/01.alc.0000080670.59386.6e

28 Flannery BA, Poole SA, Gallop RJ, et al. Alcohol craving predicts drinking during treatment: an analysis of three assessment instruments. J Stud Alcohol 2003;64:120–6.

29 Francois C, Rahhali N, Chalem Y, et al. The effects of as-needed nalmefene on patient-reported outcomes and quality of life in relation to a reduction in alcohol consumption in alcohol-dependent patients. PLoS One Published Online First: 2015. doi:10.1371/journal.pone.0129289

30 Fucito L, DeMartini KS, Leeman RF, et al. Disturbed sleep patterns among heavy drinking young adults predict alcohol treatment outcomes. Sleep 2013;:A309.

31 Gelernter J, Gueorguieva R, Kranzler HR, et al. Opioid receptor gene (OPRM1, OPRK1, and OPRD1) variants and response to naltrexone treatment for alcohol dependence: results from the VA Cooperative Study. Alcohol Clin Exp Res 2007;31:555–63. doi:10.1111/j.1530-0277.2007.00339.x

32 Harrison TS, Plosker GL, Keam SJ. Extended-release intramuscular naltrexone. Drugs 2006;:1741–51.

33 Hu X, Weber K, Karki M, et al. A pilot study of pharmacotherapy (naltrexone) for hazardous drinking among women infected with hiv. Value Health 2013;16:A70.

34 Jaffe AJ, Rounsaville B, Chang G, et al. Naltrexone, relapse prevention, and supportive therapy with alcoholics: an analysis of patient treatment matching. J Consult Clin Psychol 1996;64:1044–53.

35 Johnson BA, O'Malley SS, Ciraulo DA, et al. Dose-ranging kinetics and behavioral pharmacology of naltrexone and acamprosate, both alone and combined, in alcohol-dependent subjects. J Clin Psychopharmacol 2003;23:281–93. doi:10.1097/01.jcp.0000084029.22282.bb

36 Kaczkurkin AN, Asnaani A, Alpert E, et al. The impact of treatment condition and the lagged effects of PTSD symptom severity and alcohol use on changes in alcohol craving. Behav Res Ther 2016;79:7–14. doi:10.1016/j.brat.2016.02.001

37 Kiefer F, Andersohn F, Otte C, et al. Long-term effects of pharmacotherapy on relapse prevention in alcohol dependence. Acta Neuropsychiatr 2004;16:233–8. doi:10.1111/j.0924-2708.2004.00093.x

38 Kiefer F, Helwig H, Tarnaske T, et al. Pharmacological relapse prevention of alcoholism: clinical predictors of outcome. Eur Addict Res 2005;11:83–91. doi:10.1159/000083037

39 Kiefer F, Jahn H, Briken P, et al. Naltrexone versus acamprosate in the relapse prevention of alcoholism: A randomized placebo controlled trial. Eur Neuropsychopharmacol 2002;12:S391.

40 King AC, Zhang L, Cao D. Heavy drinkers show more pronounced effects of naltrexone on drinking and smoking outcomes within smoking cessation treatment. Alcohol Clin Exp Res 2012;:366a.

41 King A, Cao D, Vanier C, et al. Naltrexone decreases heavy drinking rates in smoking cessation treatment: an exploratory study. Alcohol Clin Exp Res 2009;33:1044–50. doi:10.1111/j.1530-0277.2009.00925.x

42 Kranzler HR, Armeli S, Covault J, et al. Variation in OPRM1 moderates the effect of desire to drink on subsequent drinking and its attenuation by naltrexone treatment. Addict Biol 2012;18:193–201. doi:10.1111/j.1369-1600.2012.00471.x

43 Kranzler HR, Armeli S, Feinn R, et al. Targeted naltrexone treatment moderates the relations between mood and drinking behavior among problem drinkers. J Consult Clin Psychol 2004;72:317–27. doi:10.1037/0022-006x.72.2.317

44 LoCastro JS, Youngblood M, Cisler RA, et al. Alcohol treatment effects on secondary nondrinking outcomes and quality of life: the COMBINE study. J Stud Alcohol Drugs 2009;70:186–96.

45 Lucey MR, Silverman BL, Illeperuma A, et al. Hepatic safety of once-monthly injectable extended-release naltrexone administered to actively drinking alcoholics. Alcohol Clin Exp Res 2008;32:498–504. doi:10.1111/j.1530-0277.2007.00593.x

46 Martin PR, Loewy J, Liou S, et al. Correlation of Serum Gamma-Glutamyl Transferase With Alcohol Consumption. 158th Annu Meet Am Psychiatr Assoc 2005 May 21-26 Atlanta GA 2005;:Nr215.

47 Miller AC, Livchits V, Atwood S, et al. Gender differences in effect of reduction in alcohol use on hiv risk behavior in tomsk, russian federation. Am J Epidemiol 2013;:S171. doi:10.1093/aje/kwt103

48 O'Malley S. Naltrexone for heavy drinking in young adults. Alcohol Clin Exp Res 2012;:334a. doi:10.1111/j.1530-0277.2012.01804.x

49 O'Malley SS, Jaffe AJ, Chang G, et al. Six-month follow-up of naltrexone and psychotherapy for alcohol dependence. Arch Gen Psychiatry 1996;53:217–24.

50 Oslin DW, Berrettini W, Kranzler HR, et al. A functional polymorphism of the mu-opioid receptor gene is associated with naltrexone response in alcohol-dependent patients. Neuropsychopharmacology 2003;28:1546–52. doi:10.1038/sj.npp.1300219

51 Oslin DW, O'Brien CP, Berrettini W, et al. Prospective study of Asn40Asp as a moderator of naltrexone treatment of alcohol dependence. Alcohol Clin Exp Res 2014;:327a.

52 Oslin DW, Pettinati H, Volpicelli JR. Alcoholism treatment adherence: older age predicts better adherence and drinking outcomes. Am J Geriatr Psychiatry 2002;10:740–7.

53 Peters EN, Leeman RF, Fucito LM, et al. Co-occurring marijuana use is associated with medication nonadherence and nonplanning impulsivity in young adult heavy drinkers. Addict Behav 2011;37:420–6. doi:10.1016/j.addbeh.2011.11.036

54 Petrakis IL, Poling J, Levinson C, et al. Naltrexone and disulfiram in patients with alcohol dependence and comorbid post-traumatic stress disorder. Biol Psychiatry 2006;60:777–83. doi:10.1016/j.biopsych.2006.03.074

55 Petrakis I, Ralevski E, Nich C, et al. Naltrexone and disulfiram in patients with alcohol dependence and current depression. J Clin Psychopharmacol 2007;27:160–5. doi:10.1097/jcp.0b13e3180337fcb

56 Pettinati HM, Gastfriend DR, Dong Q, et al. Effect of extended-release naltrexone (XR-NTX) on quality of life in alcohol-dependent patients. Alcohol Clin Exp Res 2008;33:350–6. doi:10.1111/j.1530-0277.2008.00843.x

57 Pettinati HM, Volpicelli JR, Pierce JD Jr, et al. Improving naltrexone response: an intervention for medical practitioners to enhance medication compliance in alcohol dependent patients. J Addict Dis 2000;19:71–83. doi:10.1300/J069v19n01_06

58 Ralevski E, Balachandra K, Gueorguieva R, et al. Effects of naltrexone on cognition in a treatment study of patients with schizophrenia and comorbid alcohol dependence. J Dual Diagn 2006;2:53–69. doi:10.1300/J374v02n04_05

59 Ralevski E, Ball S, Nich C, et al. The impact of personality disorders on alcohol-use outcomes in a pharmacotherapy trial for alcohol dependence and comorbid Axis I disorders. Am J Addict 2007;16:443–9. doi:10.1080/10550490701643336

60 Ralevski E, Gianoli MO, McCarthy E, et al. Quality of life in veterans with alcohol dependence and co-occurring mental illness. Addict Behav 2013;39:386–91. doi:10.1016/j.addbeh.2013.06.002

61 Richardson K, Baillie A, Reid S, et al. Do acamprosate or naltrexone have an effect on daily drinking by reducing craving for alcohol? Addiction 2008;103:953–9. doi:10.1111/j.1360-0443.2008.02215.x

62 Roberts JS, Anton RF, Latham PK, et al. Factor structure and predictive validity of the Obsessive Compulsive Drinking Scale. Alcohol Clin Exp Res 1999;23:1484–91.

63 Rohsenow DJ, Colby SM, Monti PM, et al. Predictors of compliance with naltrexone among alcoholics. Alcohol Clin Exp Res 2000;24:1542–9.

64 Rounsaville BJ, Carroll KM, Fenton LR. Enhancing naltrexone treatment after detoxification. Proc 151st Annu Meet Am Psychiatr Assoc 1998 30th May-4th June Tor Ont Can Published Online First: 1998.http://onlinelibrary.wiley.com/o/cochrane/clcentral/articles/959/CN-00713959/frame.html

65 Rybakowski JK, Volpicelli JR, Ziolkowski M. Drinking relapse in male alcoholics during treatment with naltrexone, lithium or carbamazepine. 152nd Annu Meet Am Psychiatr Assoc Wash DC USA 15-20th May 1999 Published Online First: 1999.http://onlinelibrary.wiley.com/o/cochrane/clcentral/articles/207/CN-00284207/frame.html

66 Schaumberg K, Kuerbis A, Morgenstern J, et al. Attributions of change and self-efficacy in a randomized controlled trial of medication and psychotherapy for problem drinking. Behav Ther 2013;44:88–99. doi:10.1016/j.beth.2012.07.001

67 Shin S, Livchits V, Connery HS, et al. Effectiveness of alcohol treatment interventions integrated into routine tuberculosis care in Tomsk, Russia. Addiction 2013;108:1387–96. doi:10.1111/add.12148

68 Taintor Z, Landsberg R, Wicks N. Experiences with Naltrexone in Buffalo. Am J Drug Alcohol Abuse 1975;2:391–401.

69 Zandberg LJ, Rosenfield D, Alpert E, et al. Predictors of dropout in concurrent treatment of posttraumatic stress disorder and alcohol dependence: Rate of improvement matters. Behav Res Ther 2016;80:1–9. doi:10.1016/j.brat.2016.02.005

70 Zandberg LJ, Rosenfield D, McLean CP, et al. Concurrent treatment of posttraumatic stress disorder and alcohol dependence: Predictors and moderators of outcome. J Consult Clin Psychol 2016;84:43–56. doi:10.1037/ccp0000052

71 Ziólkowski M, Rybakowski J, Kosmowski W, et al. Psychological and clinical factors predicting alcohol abstinence during 16-weeks treatment with naltrexone. 9th Congr Assoc Eur Psychiatr Cph Den 20-24th Sept 1998 Published Online First: 1998.http://onlinelibrary.wiley.com/o/cochrane/clcentral/articles/142/CN-00286142/frame.html

List of articles providing no data to calculate an effect size, neither on consumption (*i.e.*, quantity of alcohol consumed or frequency of drinking) nor abstinence outcomes

72 Anton RF. Testing combined pharmacotherapies and behavioral interventions for alcohol dependence (the COMBINE study): A pilot feasibility study. Alcohol Clin Exp Res 2003;27:1123–31. doi:10.1097/01.alc.0000078020.92938.0b

73 Brown ES, Carmody TJ, Schmitz JM, et al. A randomized, double-blind, placebo-controlled pilot study of naltrexone in outpatients with bipolar disorder and alcohol dependence. Alcohol Clin Exp Res 2009;33:1863–9. doi:10.1111/j.1530-0277.2009.01024.x

74 Heinala P, Alho H, Kiianmaa K, et al. Targeted use of naltrexone without prior detoxification in the treatment of alcohol dependence: a factorial double-blind, placebo-controlled trial. J Clin Psychopharmacol 2001;21:287–92.

75 Huang MC, Chen CH, Yu JM, et al. A double-blind, placebo-controlled study of naltrexone in the treatment of alcohol dependence in Taiwan. Addict Biol 2005;10:289–92. doi:10.1080/13556210500223504

76 Kampman KM, Pettinati HM, Lynch KG, et al. Modafinil and naltrexone for the treatment of comorbid cocaine and alcohol dependence. Drug Alcohol Depend 2015;:e152. doi:10.1016/j.drugalcdep.2014.09.331

77 Kiefer F, Jahn H, Tarnaske T, et al. Comparing and combining naltrexone and acamprosate in relapse prevention of alcoholism: a double-blind, placebo-controlled study. Arch Gen Psychiatry 2003;60:92–9.

78 Knox PC, Donovan DM. Using naltrexone in inpatient alcoholism treatment. J Psychoact Drugs 1999;31:373–88. doi:10.1080/02791072.1999.10471767

79 Kranzler HR, Armeli S, Tennen H, et al. Targeted naltrexone for early problem drinkers. J Clin Psychopharmacol 2003;23:294–304. doi:10.1097/01.jcp.0000084030.22282.6d

80 Mann K, Lemenager T, Hoffmann S, et al. Results of a double-blind, placebo-controlled pharmacotherapy trial in alcoholism conducted in Germany and comparison with the US COMBINE study. Addict Biol 2013;18:937–46. doi:10.1111/adb.12012

81 Monterosso JR, Flannery BA, Pettinati HM, et al. Predicting treatment response to naltrexone: the influence of craving and family history. Am J Addict 2001;10:258–68.

82 Monti PM, Rohsenow DJ, Swift RM, et al. Naltrexone and cue exposure with coping and communication skills training for alcoholics: treatment process and 1-year outcomes. Alcohol Clin Exp Res 2001;25:1634–47.

83 NCT00501631. ALK21-014: Efficacy and Safety of Medisorb® Naltrexone (VIVITROL®) After Enforced Abstinence. 2011.

84 Niederhofer H, Staffen W, Mair A. Comparison of naltrexone and placebo in treatment of alcohol dependence of adolescents. Alcohol Treat Q 2003;21:87–95. doi:10.1300/J020v21n02_06

85 O'Malley SS, Sinha R, Grilo CM, et al. Naltrexone and cognitive behavioral coping skills therapy for the treatment of alcohol drinking and eating disorder features in alcohol-dependent women: a randomized controlled trial. Alcohol Clin Exp Res 2007;31:625–34. doi:10.1111/j.1530-0277.2007.00347.x

86 Oslin D, Liberto JG, O'Brien J, et al. Naltrexone as an adjunctive treatment for older patients with alcohol dependence. Am J Geriatr Psychiatry 1997;5:324–32.

87 Oslin DW. Treatment of late-life depression complicated by alcohol dependence. Am J Geriatr Psychiatry 2005;13:491–500. doi:10.1176/appi.ajgp.13.6.491

88 Petrakis IL, Ralevski E, Desai N, et al. Noradrenergic vs serotonergic antidepressant with or without naltrexone for veterans with PTSD and comorbid alcohol dependence. Neuropsychopharmacology 2012;37:996–1004. doi:10.1038/npp.2011.283

89 Pettinati HM, Kampman KM, Lynch KG, et al. Gender differences with high-dose naltrexone in patients with co-occurring cocaine and alcohol dependence. J Subst Abuse Treat 2008;34:378–90. doi:10.1016/j.jsat.2007.05.011

90 Schmitz JM, Lindsay JA, Green CE, et al. High-dose naltrexone therapy for cocaine-alcohol dependence. Am J Addict 2009;18:356–62. doi:10.3109/10550490903077929

91 Schmitz JM, Stotts AL, Sayre SL, et al. Treatment of cocaine-alcohol dependence with naltrexone and relapse prevention therapy. Am J Addict 2004;13:333–41. doi:10.1080/10550490490480982

Additional file 1: Table S1. Main characteristics of the included studies

Study	Published in a medical journal	Treatment duration	Medical condition	Abstinence ≥ 5 days required	Systematic somatic or psychiatric comorbidity	Gender	Experimental treatment	Galenic form	Dose	Psychological support	Quality rating	Number of patients randomized	Outcomes reported
Anton et al.2004 [35]	YES	12	AD	NO	NO	Mixed	NLM	per os	20 mg/day<br>40 mg/day (MAX)	YES	IOD: HR<br>SOR: LR	NLM: 66<br>(MAX: 68)<br>PLB: 68	QoA<br>FoD<br>Abstinence
CPH-101-0399†	NO	16	AUD	NO	NO	Mixed	NLM	per os	40mg/day	YES	IOD: LR<br>SOR: LR	NLM: 50<br>PLB: 50	QoA<br>FoD<br>Abstinence
CPH-101-0701†	NO	28	AUD	NO	NO	Mixed	NLM	per os	10 to 40 mg/day<br>(as needed)	NO	IOD: HR<br>SOR: LR	NLM: 85<br>PLB: 82	QoA<br>FoD<br>Abstinence
Gual et al. 2013 [36]	YES	24	AD	NO	NO	Mixed	NLM	per os	20 mg/day<br>(as needed)	YES	IOD: HR<br>SOR: LR	NLM: 358<br>PLB: 360	QoA<br>FoD<br>Abstinence
Karhuvaara et al. 2007 [37]	YES	28	AUD	NO	NO	Mixed	NLM	per os	10 to 40 mg/day<br>(as needed)	YES	IOD: HR<br>SOR: LR	NLM: 242<br>PLB: 161	QoA<br>FoD<br>Abstinence
Mann et al. 2013 [38]	YES	24	AD	NO	NO	Mixed	NLM	per os	20 mg/day<br>(as needed)	YES	IOD: HR<br>SOR: LR	NLM: 306<br>PLB: 298	QoA<br>FoD<br>Abstinence
Mason et al. 1994 [39]	YES	12	AD	NO	NO	Mixed	NLM	per os	20 mg twice daily	YES	IOD: UR<br>SOR: HR	NLM: 7<br>PLB: 7	QoA<br>Abstinence
Mason et al. 1999 [40]	YES	12	AD	NO	NO	Mixed	NLM	per os	10 or 40 mg twice daily	YES	IOD: UR<br>SOR: HR	NLM: 70<br>PLB: 35	QoA<br>Abstinence
Van den Brink et al. 2014 [41]	YES	52	AD	NO	NO	Mixed	NLM	per os	20mg/day<br>(as needed)	YES	IOD: HR<br>SOR: LR	NLM: 509<br>PLB: 166	QoA<br>FoD<br>Abstinence
Ahmadi et al. 2004 [42]	YES	36	AD	NO	NO	Men only	NTX	per os	50 mg/day	YES	IOD: HR<br>SOR: UR	NTX: 58<br>PLB: 58	Abstinence
Anton et al. 1999 [43]	YES	12	AD	YES	NO	Mixed	NTX	per os	50 mg/day	YES	IOD: LR<br>SOR: UR	NTX: 68<br>PLB: 63	QoA<br>Abstinence
Anton et al. 2005 [44]	YES	12	AD	YES	NO	Mixed	NTX	per os	50 mg/day	YES	IOD: HR<br>SOR: UR	NTX: 80*<br>PLB: 80*	QoA<br>Abstinence
Anton et al. 2006 [45]	YES	16	AD	NO	NO	Mixed	NTX	per os	100 mg/day	YES	IOD: HR<br>SOR: LR	NTX: 309<br>PLB: 309	Abstinence
Anton et al. 2011a [46]	YES	16	AD	NO	NO	Mixed	NTX	per os	50 mg/day	YES	IOD: LR<br>SOR: LR	NTX: 50<br>PLB: 50	QoA<br>FoD
Anton et al. 2011b (NCT00667875)‡	NO	16	AD	NO	NO	Mixed	NTX	per os	50 mg/day	YES	IOD: HR<br>SOR: HR	NTX: 21<br>PLB: 23	QoA

Study	Published in a medical journal	Treatment duration	Medical condition	Abstinence ≥ 5 days required	Systematic somatic or psychiatric comorbidity	Gender	Experimental treatment	Galenic form	Dose	Psychological support	Quality rating	Number of patients randomized	Outcomes reported
Balldin et al. 2003 [47]	YES	24	AD	YES	NO	Mixed	NTX	per os	50 mg/day	YES	IOD: LR<br>SOR: UR	NTX: 56<br>PLB: 62	QoA<br>FoD
Baltieri et al. 2008 [48]	YES	12	AD	YES	NO	Men only	NTX	per os	50 mg/day	YES	IOD: HR<br>SOR: UR	NTX: 49<br>PLB: 54	FoD<br>Abstinence
Budzynski et al. 2000 [49]	YES	16	AD	YES	NO	Men only	NTX	per os	50 mg/day	YES	IOD: UR<br>SOR: UR	NTX: 41<br>PLB: 40	Abstinence
Castro et al. 2009 [50]	YES	12	AD	YES	NO	Mixed	NTX	per os	50 mg/day	YES	IOD: UR<br>SOR: UR	NTX: 35<br>PLB: 36	FoD
Chick et al. 2000 [51]	YES	12	AUD	YES	NO	Mixed	NTX	per os	50 mg/day	YES	IOD: HR<br>SOR: UR	NTX: 90<br>PLB: 85	QoA<br>FoD<br>Abstinence
Cook et al. 2017a [52]	YES	16	AUD	NO	YES (HIV)	Women only	NTX	per os	50 mg/day	YES	IOD: HR<br>SOR: LR	NTX: 10<br>PLB: 7	QoA<br>FoD<br>Abstinence
Cook et al. 2017b (NCT01625091)‡	NO	16	AUD	NO	YES (HIV)	Women only	NTX	per os	50 mg/day	YES	IOR : HR<br>SOR : LR	NTX : 96<br>PLB : 98	FoD<br>Abstinence
Davidson et al. 2004 [53]	YES	10	AUD	NO	NO	Mixed	NTX	per os	50 mg/day	YES	IOD: UR<br>SOR: HR	NTX: 22<br>PLB: 19	QoA<br>FoD<br>Abstinence
Foa et al. 2013 [54]	YES	24	AD	NO	YES (PTSD)	Mixed	NTX	per os	100 mg/day	YES	IOD: HR<br>SOR: LR	NTX: 82<br>PLB: 83	FoD
Fogaça et al. 2011 [55]	YES	13	AD	YES	NO	Men only	NTX	per os	50 mg/day	NO	IOD: HR<br>SOR: LR	NTX: 20<br>PLB: 20	FoD
Fridberg et al. 2014 [56]	YES	13	AUD	NO	YES (smoking)	Mixed	NTX	per os	50 mg/day	YES	IOD: HR<br>SOR: UR	NTX: 161<br>PLB: 154	QoA
Garbutt et al. 2005 [57]	YES	24	AD	NO	NO	Mixed	NTX	im	380 mg every month	YES	IOD: HR<br>SOR: LR	NTX: 208<br>PLB: 209	FoD<br>Abstinence
Garbutt et al. 2016 [58]	YES	12	AD	NO	NO	Mixed	NTX	per os	50 mg/day	YES	IOD: HR<br>SOR: LR	NTX: 40<br>PLB: 40	FoD<br>Abstinence
Gastpar et al. 2002 [59]	YES	12	AUD	YES	NO	Mixed	NTX	per os	50 mg/day	YES	IOD: HR<br>SOR: UR	NTX: 84<br>PLB: 87	Abstinence
Guardia et al. 2002 [60]	YES	12	AD	YES	NO	Mixed	NTX	per os	50 mg/day	YES	IOD: HR<br>SOR: UR	NTX: 101<br>PLB: 101	Abstinence
Hersh et al. 1998 [61]	YES	7	AUD	NO	YES (cocaine)	Mixed	NTX	per os	50 mg/day	YES	IOD: HR<br>SOR: UR	NTX: 31<br>PLB: 33	QoA<br>FoD

Study	Published in a medical journal	Treatment duration	Medical condition	Abstinence ≥ 5 days required	Systematic somatic or psychiatric comorbidity	Gender	Experimental treatment	Galenic form	Dose	Psychological support	Quality rating	Number of patients randomized	Outcomes reported
Johnson et al. 2004 [62]	YES	16	AD	YES	NO	Mixed	NTX	im	400 mg every month	YES	IOD: HR SOR: UR	NTX: 25 PLB: 5	QoA FoD Abstinence
Killeen et al. 2004 [63]	YES	12	AUD	NO	NO	Mixed	NTX	per os	50 mg/day	YES	IOD: HR SOR: UR	NTX: 54 PLB: 43	QoA FoD
Kranzler et al. 2000 [64]	YES	11	AD	NO	NO	Mixed	NTX	per os	50 mg/day	YES	IOD: HR SOR: UR	NTX: 61 PLB: 63	QoA FoD Abstinence
Kranzler et al. 2004 [65]	YES	12	AD	NO	NO	Mixed	NTX	im	300 mg the first month then 150 mg monthly	YES	IOD: HR SOR: UR	NTX: 158 PLB: 157	FoD Abstinence
Kranzler et al. 2009 [66]	YES	12	AUD	NO	NO	Mixed	NTX	per os	50 mg/day	YES	IOD: HR SOR: HR	NTX: 45 PLB: 41	QoA
Krystal et al. 2001 [67]	YES	52	AD	YES	NO	Mixed	NTX	per os	50 mg/day	YES	IOD: HR SOR: UR	NTX: 209 PLB: 209	QoA FoD
Latt et al. 2002 [68]	YES	12	AD	YES	NO	Mixed	NTX	per os	50 mg/day	NO	IOD: HR SOR: UR	NTX: 56 PLB: 51	QoA FoD Abstinence
Lee et al. 2001 [69]	YES	12	AD	YES	NO	Men only	NTX	per os	50 mg/day	YES	IOD: HR SOR: UR	NTX: 35 PLB: 18	Abstinence
Morgenstern et al. 2012 [70]	YES	12	AUD	NO	NO	Men only	NTX	per os	100 mg/day	YES	IOD: LR SOR: UR	NTX: 51 PLB: 48	QoA FoD
Morley et al. 2006 [71]	YES	12	AUD	NO	NO	Mixed	NTX	per os	50 mg/day	YES	IOD: HR SOR: UR	NTX: 53 PLB: 61	QoA Abstinence
Morris et al. 2001 [72]	YES	12	AD	NO	NO	Men only	NTX	per os	50 mg/day	YES	IOD: HR SOR: UR	NTX: 55 PLB: 56	QoA FoD Abstinence
O'Malley et al. 1992 [73]	YES	12	AD	YES	NO	Mixed	NTX	per os	50 mg/day	YES	IOD: HR SOR: UR	NTX: 52 PLB: 52	QoA FoD Abstinence
O'Malley et al. 2008a [74]	YES	16	AD	NO	NO	Mixed	NTX	per os	50 mg/day	YES	IOD: HR SOR: UR	NTX: 34 PLB: 34	QoA FoD Abstinence
O'Malley et al. 2008b [75]	YES	6	AUD	NO	YES (smoking)	Mixed	NTX	per os	50 mg/day 100 mg/day (MAX)	NO	IOD: UR SOR: LR	NTX: 25 (MAX: 28) PLB: 26	Abstinence

Study	Published in a medical journal	Treatment duration	Medical condition	Abstinence ≥ 5 days required	Systematic somatic or psychiatric comorbidity	Gender	Experimental treatment	Galenic form	Dose	Psychological support	Quality rating	Number of patients randomized	Outcomes reported
O'Malley et al. 2015 [76]	YES	8	AUD	NO	NO	Mixed	NTX	per os	25 mg/day + 25 mg/day as needed	YES	IOD: HR SOR: LR	NTX: 70 PLB: 70	QoA FoD Abstinence
Oslin et al. 2008 [77]	YES	24	AD	NO	NO	Mixed	NTX	per os	100 mg/day	YES	IOD: HR SOR: UR	NTX: 126 PLB: 122	QoA FoD Abstinence
Oslin et al. 2015 [78]	YES	12	AD	NO	NO	Mixed	NTX	per os	50 mg/day	YES	IOD: HR SOR: LR	NTX: 111 PLB: 110	FoD
Petrakis et al. 2004 [79]	YES	12	AUD	NO	YES (schizophrenia)	Men only	NTX	per os	50 mg/day	YES	IOD: HR SOR: UR	NTX: 16 PLB: 15	QoA FoD
Petrakis et al. 2005 [80]	YES	12	AD	NO	YES (axis I disorder)	Mixed	NTX	per os	50 mg/day	YES	IOD: HR SOR: UR	NTX: 59 PLB: 64	FoD Abstinence
Pettinati et al. 2008 [81]	YES	11	AD	NO	YES (cocaine)	Mixed	NTX	per os	100 mg/day	YES	IOD: LR SOR: UR	NTX: 52 PLB: 54	FoD
Pettinati et al. 2010 [82]	YES	14	AD	NO	YES (depression)	Mixed	NTX	per os	100 mg/day	YES	IOD: HR SOR: HR	NTX: 49 PLB: 39	Abstinence
Pettinati et al. 2014 [83]	YES	8	AD	NO	YES (cocaine)	Mixed	NTX	im	380 mg every month	YES	IOD: LR SOR: LR	NTX: 39 PLB: 41	QoA FoD Abstinence
Salloum et al. 2014 (NCT00302133)‡	NO	12	AD	NO	YES (bipolar disorder)	Mixed	NTX	per os	50 mg/day	YES	IOD: UR SOR: LR	NTX: 9 PLB: 9	QoA Abstinence
Santos et al. 2016 [84]	YES	8	AUD	NO	YES (metamphetamine)	Men only	NTX	per os	50 mg/day as needed	YES	IOD: LR SOR: LR	NTX: 15 PLB: 15	QoA FoD
Springer et al. 2017 [85]	YES	24	AUD	NO	YES (HIV)	Mixed	NTX	im	380 mg every month	YES	IOD: HR SOR : LR	NTX : 67 PLB : 33	QoA FoD
Tidey et al. 2008 [86]	YES	3	AUD	NO	NO	Mixed	NTX	per os	50 mg/day	NO	IOD: HR SOR: HR	NTX: 88* PLB: 85*	QoA FoD
Toneatto et al. 2009 [87]	YES	11	AUD	NO	YES (pathological gambling)	Mixed	NTX	per os	50 to 250 mg/day	YES	IOD: LR SOR: LR	NTX: 27 PLB: 25	QoA FoD Abstinence
Volpicelli et al. 1992 [88]	YES	12	AD	YES	NO	Men only	NTX	per os	50 mg/day	YES	IOD: HR SOR: UR	NTX: 35* PLB: 35*	Abstinence
Volpicelli et al. 1997 [89]	YES	12	AD	YES	NO	Mixed	NTX	per os	50 mg/day	YES	IOD: HR SOR: UR	NTX: 48 PLB: 49	FoD Abstinence

† Unpublished studies provided by the EMA
‡ Unpublished studies (or partially presented at a congress), results available on Clinicaltrials.gov
* Number of patients analyzed
AD = alcohol dependence; AUD = alcohol use disorder;
NLM = nalmefene; NTX = naltrexone;
im = intramuscular;
IOD = incomplete outcome data; SOR = selective outcome reporting; LR = low risk; UR = unclear risk; HR = high risk;
QoA = quantity of alcohol consumed; FoD = frequency of drinking;
PTSD = post-traumatic stress disorder.

Additional file 1: Figure S1. Quality evaluation of studies included according to the Cochrane Collaboration tool for assessing risk of bias

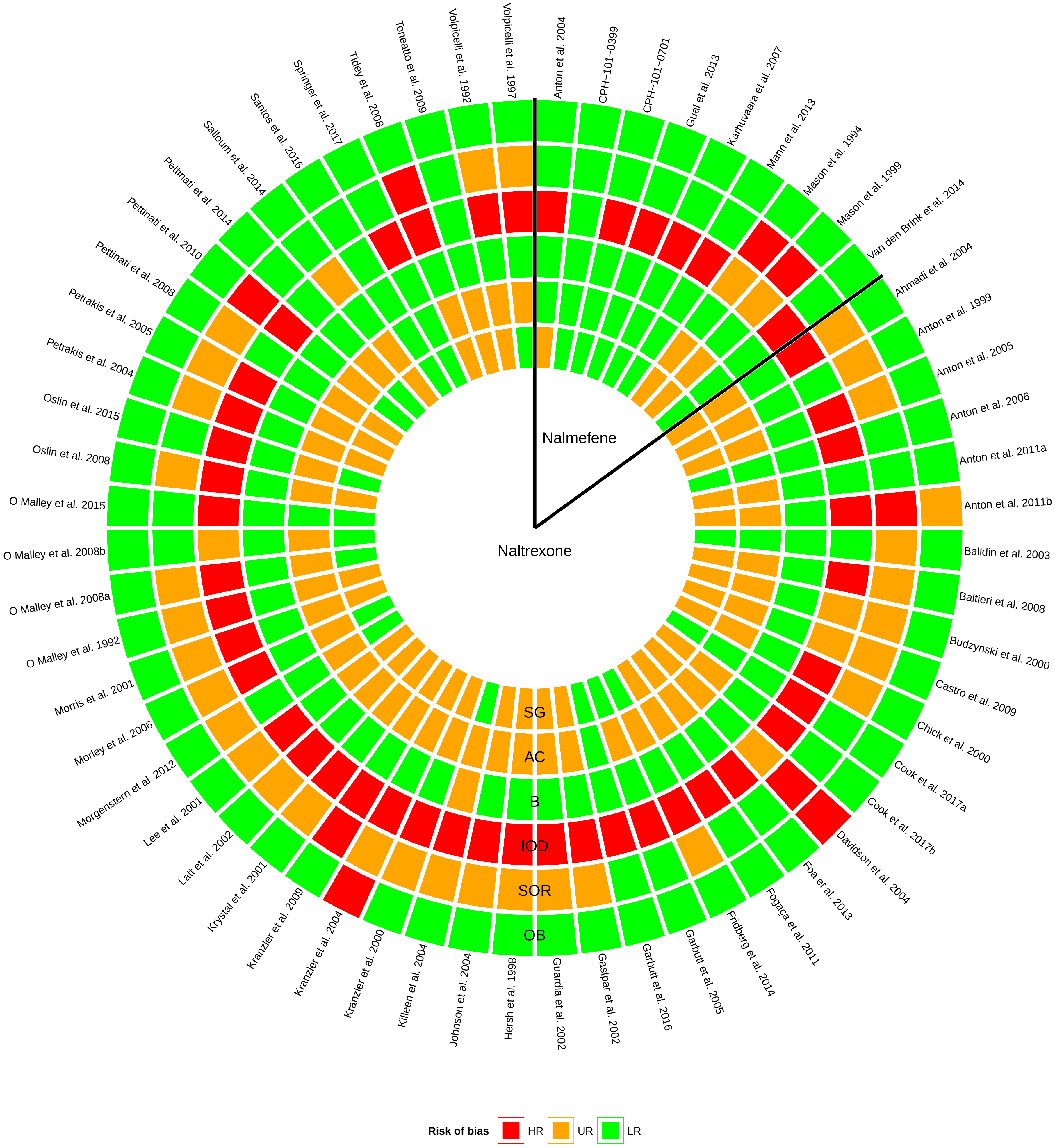
Nalmefene
Naltrexone
Anton et al. 2004
CPH−101−0399
CPH−101−0701
Gual et al. 2013
Karhuvaara et al. 2007
Mann et al. 2013
Mason et al. 1994
Mason et al. 1999
Van den Brink et al. 2014
Ahmadi et al. 2004
Anton et al. 1999
Anton et al. 2005
Anton et al. 2006
Anton et al. 2011a
Anton et al. 2011b
Balldin et al. 2003
Baltieri et al. 2008
Budzynski et al. 2000
Castro et al. 2009
Chick et al. 2000
Cook et al. 2017a
Cook et al. 2017b
Davidson et al. 2004
Foa et al. 2013
Fogaça et al. 2011
Fridberg et al. 2014
Garbutt et al. 2005
Garbutt et al. 2016
Gastpar et al. 2002
Guardia et al. 2002
Hersh et al. 1998
Johnson et al. 2004
Killeen et al. 2004
Kranzler et al. 2000
Kranzler et al. 2004
Kranzler et al. 2009
Krystal et al. 2001
Latt et al. 2002
Lee et al. 2001
Morgenstern et al. 2012
Morley et al. 2006
Morris et al. 2001
O Malley et al. 1992
O Malley et al. 2008a
O Malley et al. 2008b
O Malley et al. 2015
Oslin et al. 2008
Oslin et al. 2015
Petrakis et al. 2004
Petrakis et al. 2005
Pettinati et al. 2008
Pettinati et al. 2010
Pettinati et al. 2014
Salloum et al. 2014
Santos et al. 2016
Springer et al. 2017
Tidey et al. 2008
Toneatto et al. 2009
Volpicelli et al. 1992
Volpicelli et al. 1997
SG
AC
B
IOD
SOR
OB
Risk of bias
HR
UR
LR

SG = sequence generation;
AC = allocation concealment;
B = blinding;
IOD = incomplete outcome data;
SOR = selective outcome reporting;
OB = other bias.
For each item, the risk of bias was classified as ‘low risk’ (LR), ‘high risk’ (HR) or ‘unclear risk’ (UR), with the last category indicating either lack of information or uncertainty over the potential for bias.

Additional file 1: Table S2. Analytical scenario resulting in superiority of nalmefene over naltrexone

Category	Criteria
Medical condition	Exclusion of studies including patients with AUDs
Abstinence	Exclusion of studies requiring a minimum period of abstinence of 5 days or more before the beginning of the study
Gender	Exclusion of studies with males or females only
Somatic comorbidity	Exclusion of studies on patients with systematic somatic comorbidities (e.g. studies on patients with HIV)
Psychiatric comorbidity	Exclusion of studies on patients with systematic psychiatric comorbidities (e.g. studies on depressed patients)
Psychological support	Inclusion of all studies (with or without psychological intervention)
Treatment and dose	Only approved dose and route of administration
Treatment duration	Inclusion of all studies, regardless of treatment duration
Outcome	Quantity of alcohol consumed
Publication	Published and unpublished studies (e.g. study reports, ClinicalTrials.gov)
Risk of bias	Inclusion of all studies, regardless of the risk of selective outcome reporting
Analysis	Random effect model

**Nalmefene studies included (n=3)**	**Effect size [95%CI]**
Gual et al. 2013	-0.15 [-0.33 ; 0.04]
Mann et al. 2013	-0.35 [-0.56 ; -0.14]
Van den Brink et al. 2014	-0.13 [-0.36 ; 0.10]
**Naltrexone studies included (n=4)**	
Anton et al. 2011a	-0.13 [-0.53 ; 0.26]
Anton et al. 2011b	0.08 [-0.60 ; 0.76]
Kranzler et al. 2000	0.34 [-0.02 ; 0.69]
O'Malley et al. 2008a	-0.06 [-0.53 ; 0.41]
**Nalmefene versus naltrexone indirect comparison**	-0.29 [-0.56 ; -0.02], p = 0.037
**Heterogeneity in the network**	I^2 = 19.4%<br>Cochran's Q-test p-value= 0.287

Additional file 1: Table S3. Analytical scenario resulting in superiority of naltrexone over nalmefene

Category	Criteria
Medical condition	Exclusion of studies including patients with AUDs
Abstinence	Inclusion of all studies (abstinent or non-abstinent patients)
Gender	Inclusion of all studies (mixed gender, males only or females only)
Somatic comorbidity	Exclusion of studies on patients with systematic somatic comorbidities (e.g. studies on patients with HIV)
Psychiatric comorbidity	Inclusion of all studies (patients with or without systematic psychiatric comorbidities)
Psychological support	Exclusion of studies with no psychological intervention
Treatment and dose	Only approved dose and route of administration
Treatment duration	Exclusion of studies with a treatment duration of less than 12 weeks
Outcome	Abstinence
Publication	Published and unpublished studies (e.g. study reports, ClinicalTrials.gov)
Risk of bias	Exclusion of studies with a high risk of selective outcome reporting
Analysis	Random effect model

**Nalmefene studies included (n=3)**	**Effect size [95%CI]**
Gual et al. 2013	-0.07 [-0.25 ; 0.12]
Mann et al. 2013	-0.16 [-0.37 ; 0.04]
Van den Brink et al. 2014	0.01 [-0.20 ; 0.23]
**Naltrexone studies included (n=15)**	
Ahmadi et al. 2004	-0.46 [-0.89 ; -0.03]
Anton et al. 1999	-0.38 [-0.72 ; -0.04]
Anton et al. 2005	-0.25 [-0.56 ; 0.06]
Baltieri et al. 2008	-0.02 [-0.49 ; 0.45]
Budzynski et al. 2000	-0.13 [-0.68 ; 0.43]
Garbutt et al. 2016	-0.18 [-0.62 ; 0.25]
Guardia et al. 2002	-0.54 [-1.03 ; -0.06]
Lee et al. 2001	-0.45 [-1.16 ; 0.27]
Morris et al. 2001	-0.36 [-1.07 ; 0.35]
O'Malley et al. 1992	-0.63 [-1.12 ; -0.13]
O'Malley et al. 2008a	-0.51 [-0.99 ; -0.03]
Petrakis et al. 2005	-0.15 [-0.50 ; 0.21]
Salloum et al. 2014	-0.73 [-1.76 ; 0.30]
Volpicelli et al. 1992	-0.76 [-1.32 ; -0.19]
Volpicelli et al. 1997	-0.40 [-0.84 ; 0.05]
**Nalmefene versus naltrexone indirect comparison**	0.27 [0.11 ; 0.44], p = 0.001
**Heterogeneity in the network**	I^2 = 0.0%<br>Cochran's Q-test p-value= 0.789

Additional file 1: Figure S2. Heterogeneity of the indirect comparison: nalmefene versus naltrexone

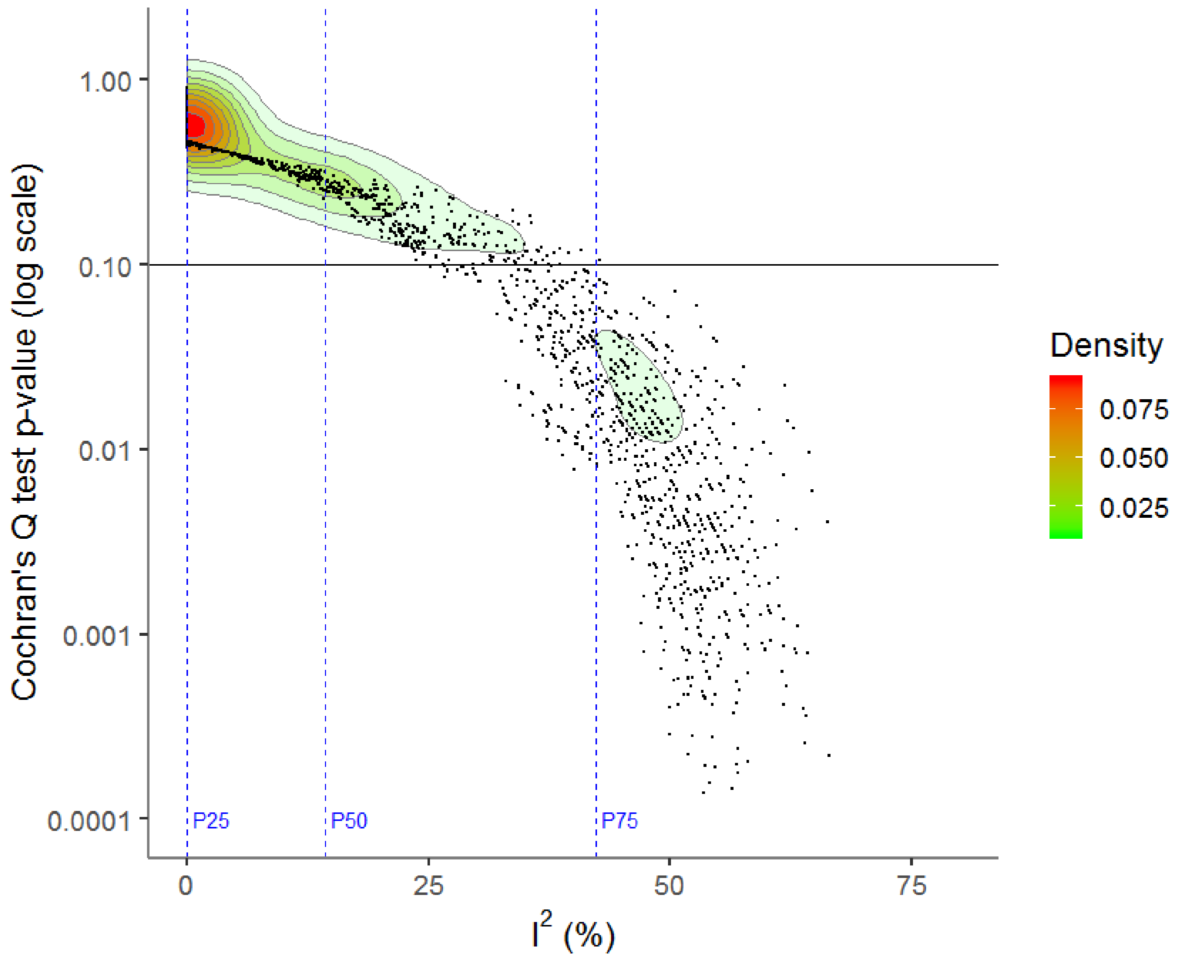

P25 = 1st quartile; P50 = median; P75 = 3rd quartile

The points represent the meta-analyses; The colours represent the densities

Additional file 1: Figure S3. Sensitivity analysis excluding meta-analyses with I2 > 25% and based on a fixed effect model for the indirect comparison between nalmefene and naltrexone

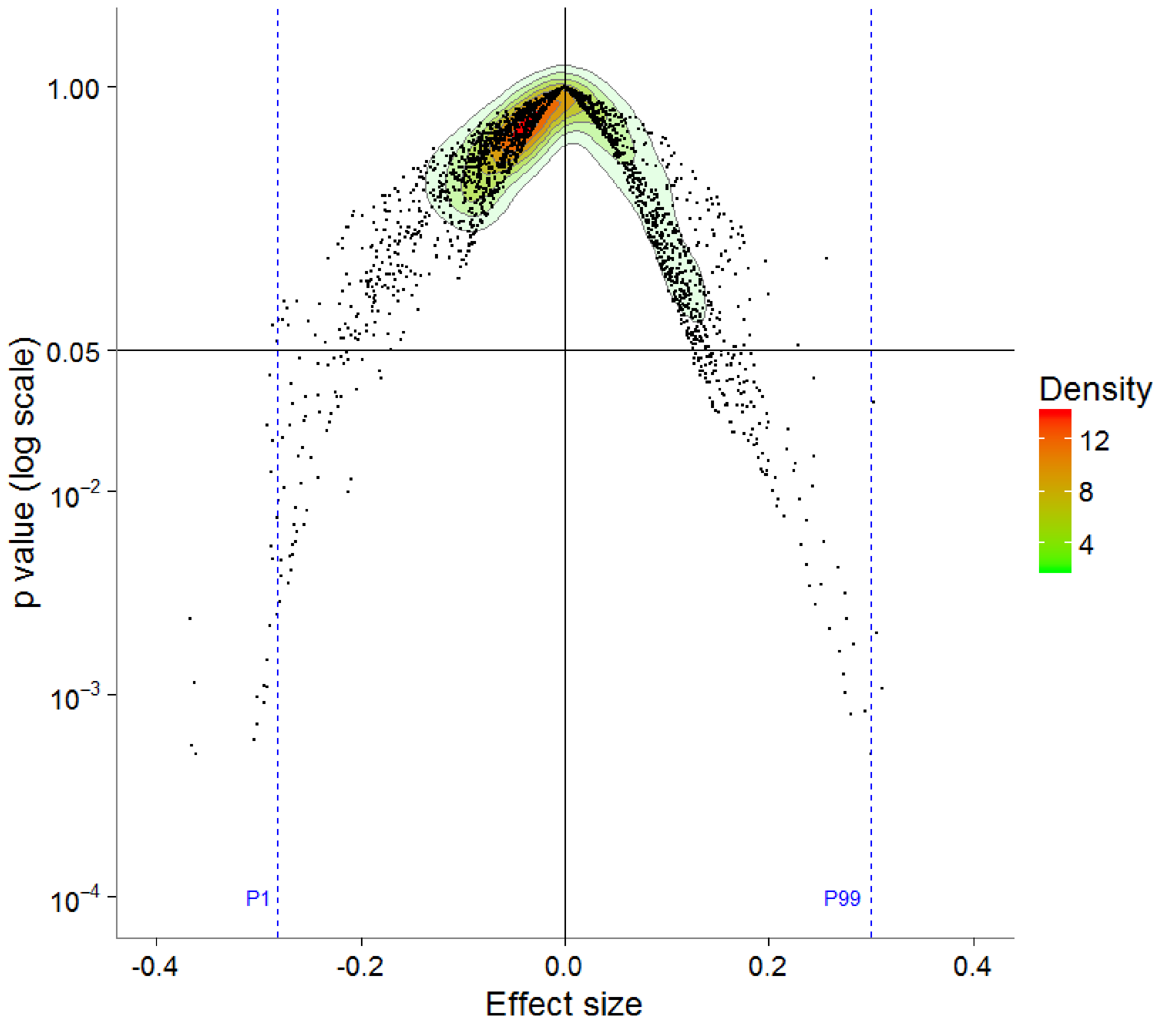

A negative effect size favours nalmefene, whereas a positive effect size favours naltrexone

The points represent the meta-analyses; The colours represent the densities

Additional file 1: Figure S4. Heterogeneity of the direct comparison: nalmefene versus placebo

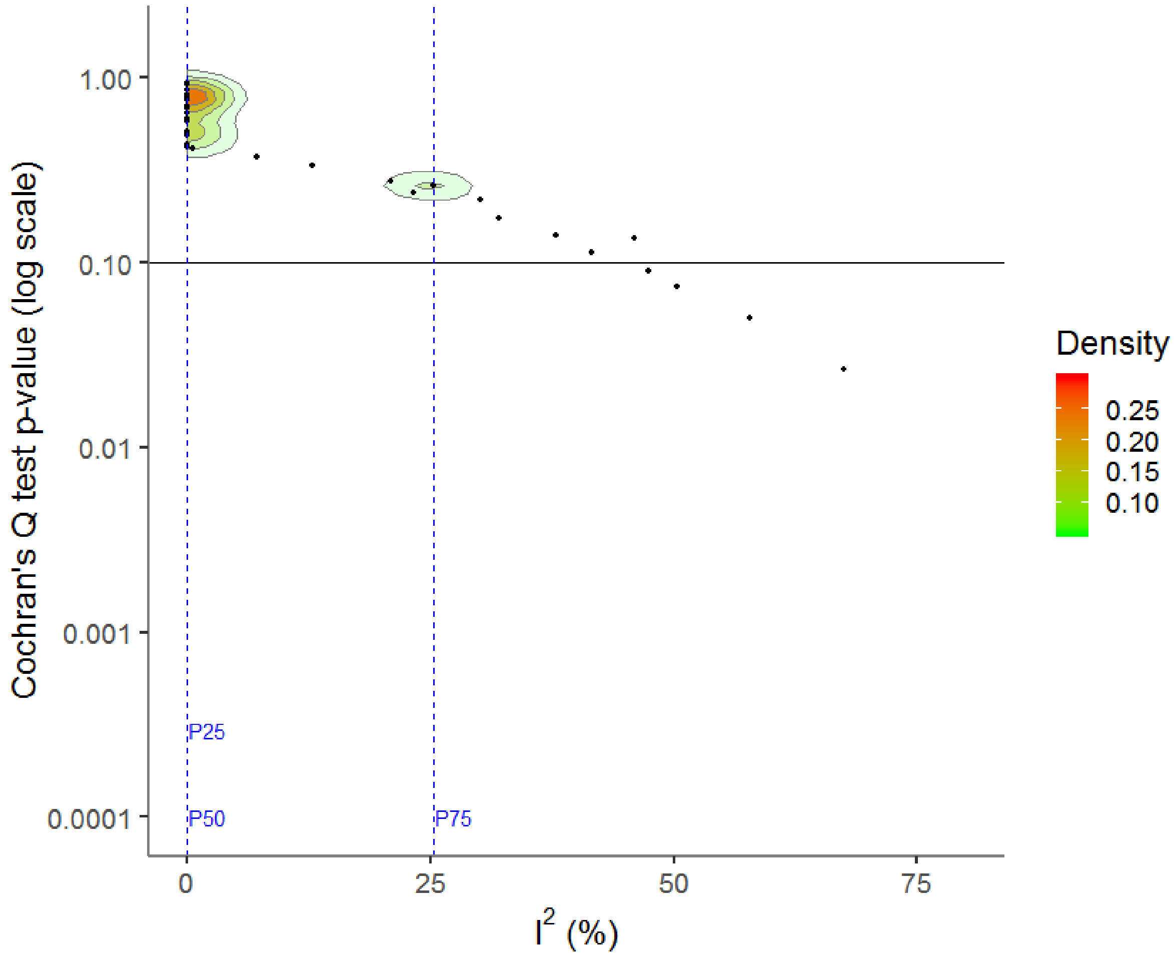

P25 = 1st quartile; P50 = median; P75 = 3rd quartile

The points represent the meta-analyses; The colours represent the densities

Additional file 1: Figure S5. Heterogeneity of the direct comparison: naltrexone versus placebo

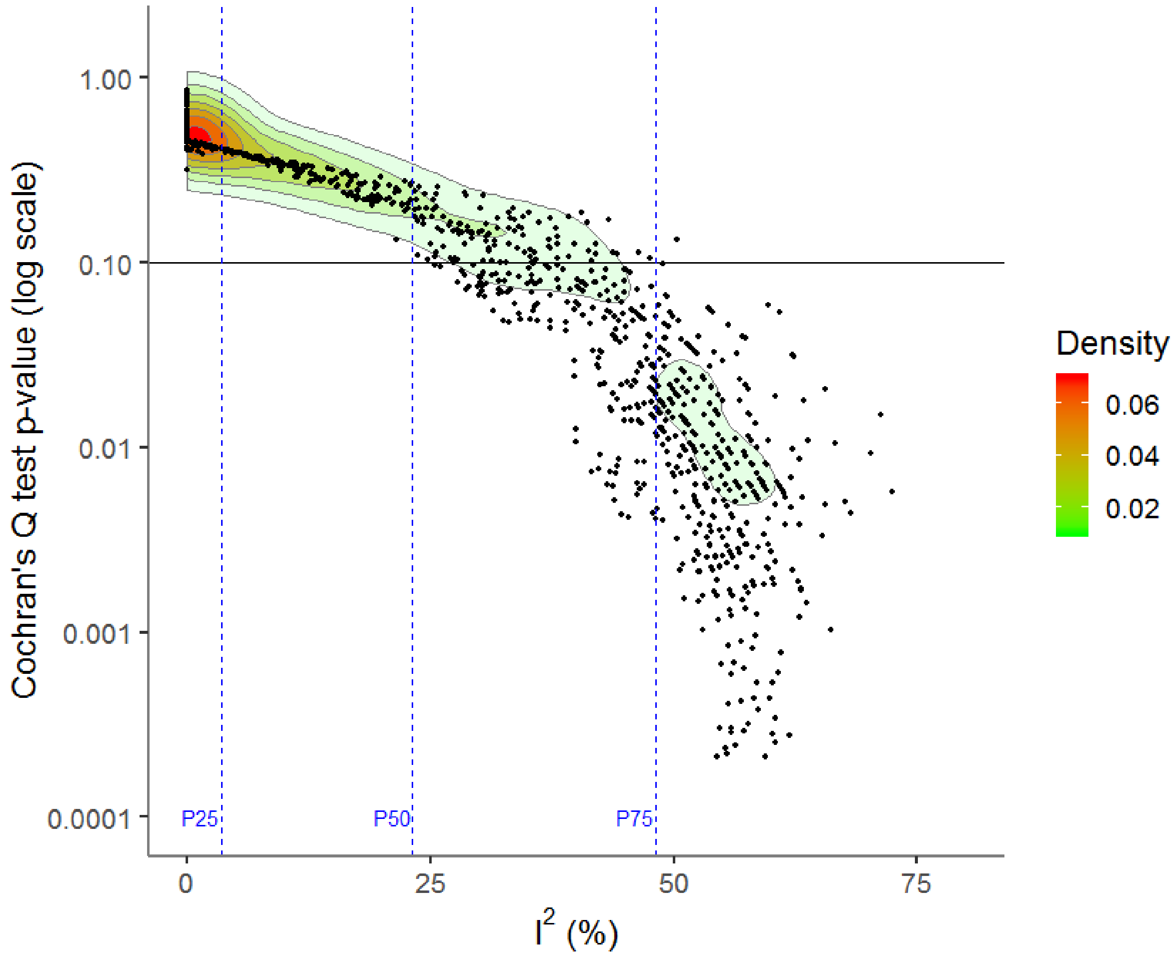

P25 = 1st quartile; P50 = median; P75 = 3rd quartile

The points represent the meta-analyses; The colours represent the densities

Additional file 1: Checklist S1. PRISMA checklist

Section/topic	#	Checklist item	Heading: Subheading
**TITLE**			
Title	1	Identify the report as a systematic review, meta-analysis, or both.	Title
**ABSTRACT**			
Structured summary	2	Provide a structured summary including, as applicable: background; objectives; data sources; study eligibility criteria, participants, and interventions; study appraisal and synthesis methods; results; limitations; conclusions and implications of key findings; systematic review registration number.	Abstract
**INTRODUCTION**			
Rationale	3	Describe the rationale for the review in the context of what is already known.	Background
Objectives	4	Provide an explicit statement of questions being addressed with reference to participants, interventions, comparisons, outcomes, and study design (PICOS).	Background
**METHODS**			
Protocol and registration	5	Indicate if a review protocol exists, if and where it can be accessed (e.g., Web address), and, if available, provide registration information including registration number.	Methods: Design
Eligibility criteria	6	Specify study characteristics (e.g., PICOS, length of follow-up) and report characteristics (e.g., years considered, language, publication status) used as criteria for eligibility, giving rationale.	Methods: Eligibility criteria
Information sources	7	Describe all information sources (e.g., databases with dates of coverage, contact with study authors to identify additional studies) in the search and date last searched.	Methods: Search strategy and study selection process
Search	8	Present full electronic search strategy for at least one database, including any limits used, such that it could be repeated.	Methods: Search strategy and study selection process
Study selection	9	State the process for selecting studies (i.e., screening, eligibility, included in systematic review, and, if applicable, included in the meta-analysis).	Methods: Search strategy and study selection process
Data collection process	10	Describe method of data extraction from reports (e.g., piloted forms, independently, in duplicate) and any processes for obtaining and confirming data from investigators.	Methods: Data collection
Data items	11	List and define all variables for which data were sought (e.g., PICOS, funding sources) and any assumptions and simplifications made.	Methods: Data collection
Risk of bias in individual studies	12	Describe methods used for assessing risk of bias of individual studies (including specification of whether this was done at the study or outcome level), and how this information is to be used in any data synthesis.	Methods: Assessment of methodological quality
Summary measures	13	State the principal summary measures (e.g., risk ratio, difference in means).	Methods: Assessment of vibration of effects
Synthesis of results	14	Describe the methods of handling data and combining results of studies, if done, including measures of consistency (e.g., I^2) for each meta-analysis.	Methods: Assessment of vibration of effects<br>Table 1

Section/topic	#	Checklist item	Heading: Subheading
Risk of bias across studies	15	Specify any assessment of risk of bias that may affect the cumulative evidence (e.g., publication bias, selective reporting within studies).	Methods: Assessment of vibration of effects
Additional analyses	16	Describe methods of additional analyses (e.g., sensitivity or subgroup analyses, meta-regression), if done, indicating which were pre-specified.	Methods: Assessment of vibration of effects
**RESULTS**			
Study selection	17	Give numbers of studies screened, assessed for eligibility, and included in the review, with reasons for exclusions at each stage, ideally with a flow diagram.	Results<br>Figure 1<br>Additional file 1: References S1
Study characteristics	18	For each study, present characteristics for which data were extracted (e.g., study size, PICOS, follow-up period) and provide the citations.	Results: Study characteristics and risk of bias within studies<br>Table 2<br>Additional file 1: Table S1
Risk of bias within studies	19	Present data on risk of bias of each study and, if available, any outcome level assessment (see item 12).	Results: Study characteristics and risk of bias within studies<br>Additional file 1: Figure S1
Results of individual studies	20	For all outcomes considered (benefits or harms), present, for each study: (a) simple summary data for each intervention group (b) effect estimates and confidence intervals, ideally with a forest plot.	Results: Vibration of effects
Synthesis of results	21	Present results of each meta-analysis done, including confidence intervals and measures of consistency.	Results: Vibration of effects<br>Figures 2-4<br>Additional file 1: Table S2-S3<br>Additional file 1: Figure S2, S4-S5
Risk of bias across studies	22	Present results of any assessment of risk of bias across studies (see Item 15).	Not performed
Additional analysis	23	Give results of additional analyses, if done (e.g., sensitivity or subgroup analyses, meta-regression [see Item 16]).	Results: Vibration of effects<br>Additional file 1: Figure S3
**DISCUSSION**			
Summary of evidence	24	Summarize the main findings including the strength of evidence for each main outcome; consider their relevance to key groups (e.g., healthcare providers, users, and policy makers).	Discussion: Statement of principal findings
Limitations	25	Discuss limitations at study and outcome level (e.g., risk of bias), and at review-level (e.g., incomplete retrieval of identified research, reporting bias).	Discussion: Strengths and weaknesses of this study<br>Discussion: Perspectives
Conclusions	26	Provide a general interpretation of the results in the context of other evidence, and implications for future research.	Conclusions
**FUNDING**			
Funding	27	Describe sources of funding for the systematic review and other support (e.g., supply of data); role of funders for the systematic review.	Funding statement

From: Moher D, Liberati A, Tetzlaff J, Altman DG, The PRISMA Group (2009). Preferred Reporting Items for Systematic Reviews and Meta-Analyses: The PRISMA Statement. PLoS Med 6(6): e1000097. doi:10.1371/journal.pmed1000097

For more information, visit: **www.prisma-statement.org**.
